# Critical Windows of Air Pollution Exposure During Spermatogenesis and Intrauterine Insemination Outcomes

**DOI:** 10.1111/ppe.70185

**Published:** 2026-08-04

**Authors:** Lindsey M. Russo, J. Richard Pilsner, Timothy P. Canty, Neil J. Perkins, Pauline Mendola, Karen C. Schliep, May Shaaban, Rachael B. Hemmert, Akanksha Singh, Allison M. Ring, James A. Vanderslice, C. Matthew Peterson, Erica B. Johnstone, Kaniz Rabeya, Carrie Nobles

**Affiliations:** ^1^ Division of General Internal Medicine Weill Cornell Medicine New York New York USA; ^2^ Department of Obstetrics and Gynecology Wayne State University School of Medicine Detroit Michigan USA; ^3^ Department of Atmospheric and Oceanic Science University of Maryland College Park Maryland USA; ^4^ Biostatistics and Bioinformatics Branch, Eunice Kennedy Shriver National Institute of Child Health and Human Development National Institutes of Health Bethesda Maryland USA; ^5^ Epidemiology and Environmental Health, School of Public Health and Health Professions University at Buffalo Buffalo New York USA; ^6^ Division of Public Health, Department of Family and Preventive Medicine University of Utah Health Salt Lake City Utah USA; ^7^ Division of Reproductive Endocrinology and Infertility, Department of Obstetrics and Gynecology University of Utah Health Salt Lake City Utah USA; ^8^ Department of Environmental Health Sciences, School of Public Health and Health Sciences University of Massachusetts Amherst Amherst Massachusetts USA

**Keywords:** air pollution, environment, infertility treatment, intrauterine insemination outcomes, spermatogenesis, subfertility

## Abstract

**Background:**

Male preconception health remains a critically under‐addressed area of family planning. While research suggests air pollution may harm semen quality, downstream impacts on pregnancy, particularly for more vulnerable infertility treatment populations, are understudied.

**Objectives:**

Among couples seeking infertility treatment, we examined the relationship between men's exposure to ambient air pollution and intrauterine insemination (IUI) cycle treatment outcomes.

**Methods:**

In the Folic Acid and Zinc Supplementation Trial (2013–2018), 592 male partners resided in the Salt Lake City area with at least one IUI cycle in 9 months of follow‐up (*n* = 1223 cycles). Residential air pollution exposure was estimated using Community Multiscale Air Quality models and averaged across the 74‐day window of spermatogenesis prior to the insemination date, as well as across developmental windows of mitosis, meiosis I‐II, spermiogenesis, and spermiation. Treatment‐cycle probabilities of hCG+ pregnancy, pregnancy loss, and livebirth were evaluated using generalised linear mixed models adjusted for age, season, income, and co‐pollutants (fine particulate matter [PM_2.5_], nitrogen dioxide [NO_2_], ozone [O_3_], and sulfur dioxide [SO_2_]).

**Results:**

In multivariable models, we observed a lower probability of pregnancy per 5 ppb increase in O_3_ and NO_2_ across the 74‐day spermatogenesis window (relative risk [RR] 0.71, 95% confidence interval [CI] 0.55, 0.93; RR 0.73, 95% CI 0.50, 1.05, respectively), with associations noted during mitosis and meiosis. PM_2.5_ and SO_2_ were less consistently associated with IUI outcomes. Findings from mixture models were similar, as were findings for air pollutants and a lower probability of live birth. No clear patterns emerged between air pollutants and pregnancy loss.

**Conclusions:**

Ambient air pollution during spermatogenesis, especially during mitosis and meiosis, was associated with a lower chance of pregnancy and live birth in an IUI treatment population. Further understanding pathways between men's air pollution exposure and pregnancy and live birth is an important future research need.

**Trial Registration:**

clinicaltrials.gov identifier: NCT01857310

## 1 Background

Growing evidence suggests that ambient air pollution may be associated with greater difficulty achieving pregnancy among natural pregnancy planners [1, 2, 3, 4, 5, 6] and couples undergoing in vitro fertilisation (IVF) [7]. However, despite evidence suggesting detrimental impacts of men's exposure to air pollution on spermatogenesis, most research has focused on susceptible windows of exposure for female partners, including exposure timed to ovulation and implantation. Semen quality plays an important role in couples' fecundity, and fine particulate matter (PM_2.5_) and nitrogen dioxide (NO_2_) are associated with declines in semen quality, including lower semen concentration, motility, and normal morphology [8, 9, 10, 11], higher DNA fragmentation [12], and changes in sperm epigenetics [13, 14]. Impacts of ambient air pollution on the fertilisation capacity of sperm may in turn impact the probability of pregnancy and live birth [15].

Air pollution may be particularly important among couples seeking infertility treatment. Prior research has focused on couples undergoing IVF, suggesting air pollution exposure during ovulation induction and implantation contributes to unsuccessful IVF treatment outcomes [7], including poorer embryo quality and fertilisation rate [16, 17]. However, the role of ambient air pollution exposure during spermatogenesis and its impacts on outcomes of intrauterine insemination (IUI) treatment, frequently used as a first‐line treatment for infertility [18, 19], has been less studied. Pregnancies achieved through IUI may be more comparable to natural pregnancies than IVF, particularly for impacts on spermatogenesis, as fertilisation takes place within the uterus [20].

We evaluated the association of men's exposure to ambient air pollution during spermatogenesis on the probability of pregnancy, pregnancy loss, and live birth during IUI cycles among couples seeking infertility treatment living in and around Salt Lake City, Utah. The Salt Lake City metropolitan area, positioned along the Wasatch Front, exceeds the EPA National Ambient Air Quality Standards (NAAQS) for both 24‐h PM_2.5_ due to winter temperature inversions and 8‐h ozone (O_3_) in summer [21, 22, 23]. This region is further impacted by its topography, point sources such as large oil refineries and other sources of sulfur dioxide [24], as well as traffic, making it vulnerable to air pollution in the United States. We hypothesised that higher ambient air pollution exposure during spermatogenesis may be associated with lower IUI treatment‐cycle probability of pregnancy, higher risk of pregnancy loss, and lower probability of live birth.

## 2 Methods

This is a secondary analysis of the Folic Acid and Zinc Supplementation Trial (FAZST, 2013–2018). The trial enrolled male partners of couples seeking fertility treatment (*n* = 2370) across 4 study sites, including Salt Lake City, Utah; Iowa City, Iowa; Chicago, Illinois; and Minneapolis, Minnesota. We restricted analysis to men enrolled in the Salt Lake City metropolitan area along the Wasatch Front (*n* = 2015) for residential geocoding and estimation of air pollution, and further to male partners of couples undergoing IUI (*n* = 592 participants, *n* = 1223 cycles, Figure S1). The FAZST trial randomised men to daily folic acid and zinc supplementation versus placebo and followed couples for up to 9 months of infertility treatment and throughout pregnancy if they became pregnant. In the parent FAZST trial, male partner randomisation to zinc and folic acid was not associated with semen quality or infertility treatment outcomes (pregnancy and live birth) [25]. Exclusion criteria included male partner age < 18 years; female partner age < 18 or > 45 years; male partner history of vasectomy without reversal, obstructive azoospermia, or ejaculatory duct obstruction; known genetic cause of male factor subfertility (e.g., Y chromosome deletions); or poorly controlled physician‐diagnosed chronic health condition (e.g., diabetes mellitus or cancer).

### 2.1 Outcomes of Intrauterine Insemination (IUI) Cycles

IUI is commonly used as a first‐line treatment in cases of unexplained infertility. Most IUI cycles incorporate ovulation induction drugs (e.g., clomiphene citrate, gonadotropins, letrozole) followed by insemination with fresh or thawed sperm into the uterus after initiation of ovulation by hCG injection [19, 26]. Information on IUI cycles occurring in the 9 months following enrollment was part of the FAZST data collection and included treatment dates and treatment outcomes (hCG+ pregnancy, pregnancy loss, and livebirth) (Figure 1). No frozen sperm was used in the observed IUI cycles. Pregnancy was assessed using serum hCG during a clinic visit approximately 15 days after insemination. Patients received a routine ultrasound at 6–7 weeks' gestation to confirm clinical pregnancy. Pregnancy loss included any loss during an IUI cycle over the 9 months of follow‐up, including hCG‐identified losses, in which detection of serum hCG > 5 mIU/mL was followed by a decline, and clinically recognised losses, defined as a loss < 20 weeks' gestation after confirmation of clinical pregnancy. Live birth was defined as the proportion of IUI cycles ending in live‐born infants.

**FIGURE 1:**
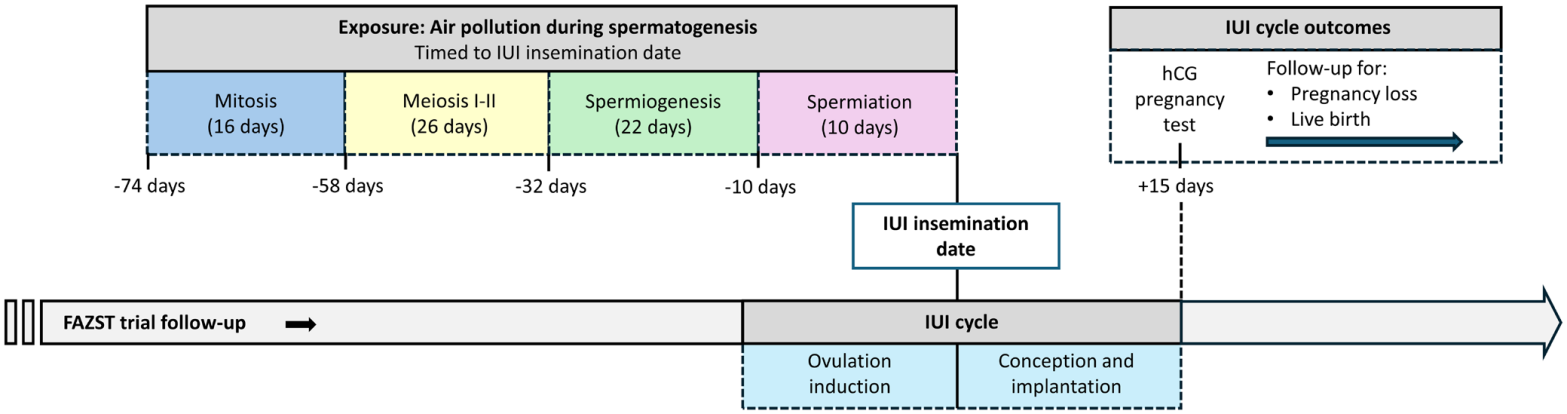
Timing of air pollution exposure windows during spermatogenesis and follow‐up for intrauterine insemination (IUI) cycle treatment outcomes for IUI cycles occurring in the FAZST trial (*n* = 1223 cycles for 592 couples during 9 months of follow‐up at the Salt Lake City study sites). All reported IUI cycles utilised fresh semen collected around the time of intrauterine insemination.

### 2.2 Air Pollution Exposure and Windows of Susceptibility Across Spermatogenesis

Daily participant exposure to PM_2.5_, sulfur dioxide (SO_2_), NO_2_, and O_3_ was estimated using Community Multiscale Air Quality (CMAQ) models, capturing daily air pollution levels (8‐h average for O_3_) at a resolution of 12 km × 12 km, with simulations provided by the National Air Quality Forecast Capability (NAQFC) Program [27]. Geocoding of FAZST study participants' residential addresses was conducted using ArcGIS software (Redlands, CA) and used to assign residential pollutant concentrations. Using the date of intrauterine insemination from clinical records for each IUI cycle, we averaged air pollution exposure across the approximate 74‐day spermatogenesis exposure window [28] leading up to the date of intrauterine insemination. We additionally estimated lookback windows for specific developmental windows of spermatogenesis (mitosis: 59–74 days prior to semen sample; meiosis I + II: 33–58 days; spermiogenesis: 11–32 days; and spermiation: 0–10 days) based on approximate dates of each developmental window during spermatogenesis as informed by the literature (Figure S2) [1]. However, uncertainty remains for the ability to completely distinguish underlying biologic windows of susceptibility for each exposure window. As a secondary analysis for comparison to prior studies, we evaluated exposure during windows corresponding to ovulation and implantation, including the 5 days leading up to ovulation (0–5 days before ovulation), pre‐implantation (1–5 days after ovulation), implantation (6–10 days after ovulation), and post‐implantation (11–14 days after ovulation).

### 2.3 Statistical Analysis

Pearson correlation coefficients demonstrated relationships among air pollutants. Windows of exposure included average air pollution across the overall 74‐day spermatogenesis window, susceptible windows of spermatogenesis, and, in secondary analysis, windows corresponding to ovulation and implantation. Three modelling approaches evaluated the relationship between air pollution and IUI treatment cycle outcomes (hCG+ pregnancy, pregnancy loss, and live birth): (i) Single‐pollutant models, (ii) Multipollutant models jointly adjusting for PM_2.5_, NO_2_, O_3_, and SO_2_, and (iii) Mixture models using quantile g‐computation. Quantile g‐computation accounts for collinearity among pollutants and overcomes limitations of traditional weighted quantile sum regression in allowing bidirectional associations of pollutants in the estimation of mixture weights [29].

For single‐ and multipollutant models, the relative risk of treatment‐cycle pregnancy, pregnancy loss, and live birth was estimated using generalised linear mixed models with a Poisson distribution and robust standard errors [30], accounting for multiple treatment cycles per couple. Quantile g‐computation evaluated the impact of a decile increase in all pollutants (PM_2.5_, NO_2_, O_3_, and SO_2_) on the relative risk of IUI treatment‐cycle outcomes [29], incorporating random intercepts to account for multiple cycles and estimating standard errors by bootstrap (200 iterations). For all models, covariates were determined a priori based on the literature and directed acyclic graphs (Figure 2), with confounders including season and area‐level structural inequities, including household income as a proxy for socioeconomic status (SES) and race/ethnicity as a proxy for exposure to structural racism. However, race/ethnicity was not retained in the final models as it did not change statistical estimates. All models adjusted for male age, season, and income. For pregnancy loss, models were restricted to hCG+ pregnancies and utilised inverse probability weighting (IPW) to address potential selection bias by accounting for pregnancy‐related factors [31].

**FIGURE 2:**
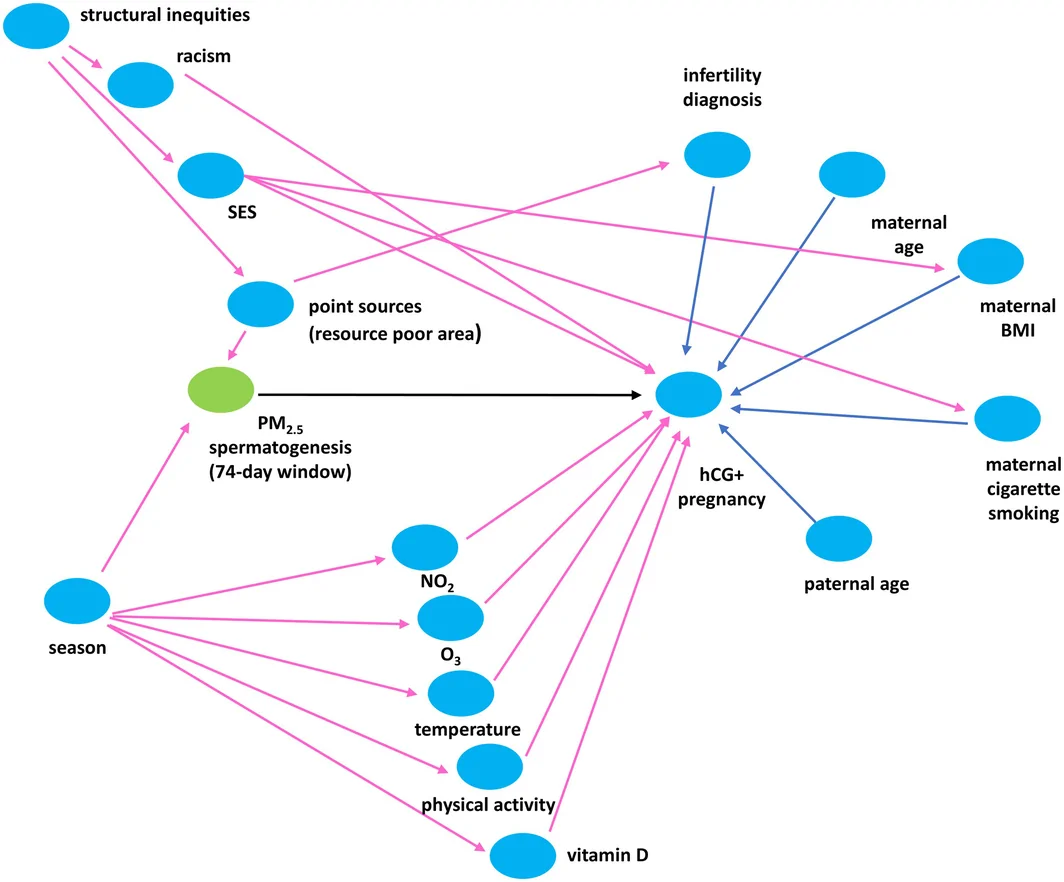
Directed acyclic graph to show proposed relation between PM_2.5_ (74‐day window) with treatment‐cycle probability of hCG+ pregnancy, and covariates.

To account for differences in air pollution mixtures by season, we evaluated effect modification for hCG+ pregnancy and live birth by warm (April–September) vs. cold (October–March) season of the IUI cycle. Although randomisation to folic acid and zinc was not associated with infertility treatment outcomes in the main trial [25], we evaluated differences by treatment assignment to account for potential antioxidant effects of folic acid and zinc. We additionally evaluated effect modification by male partner fertility status, including baseline self‐report of prior male‐factor infertility diagnosis and baseline semen quality parameters (sperm count, morphology, and motility) falling below the 5th percentile of WHO standards [32]. Counts were too small to evaluate effect modification for pregnancy loss. Additive interactions were assessed by estimating the relative excess risk due to interaction (RERI [33] with 95% confidence intervals from 1000 bootstrap samples). As female partner age [34] and infertility status [26] are strongly associated with pregnancy and live birth, we additionally evaluated both as potential confounders. Finally, we restricted analyses to the first IUI cycle of follow‐up to compare findings to the linear mixed model incorporating all IUI cycles.

Statistical analyses were conducted in SAS (version 9.4; SAS Institute Inc.) and figures created in R (version 4.4; R Development Core Team).

### 2.4 Missing Data

Among the 592 couples, baseline data were missing for 2 (0.3%) for marital status, 3 (0.5%) for female partner BMI, 20 (3.4%) for household income, 12 (2.0%) for baseline semen quality, 114 (19.3%) for self‐reported male‐factor infertility, and 115 (19.4%) for female‐factor infertility. Missing data for infertility are structural and dependent on couples having had an infertility evaluation prior to enrollment. Among the 1223 IUI cycles, data were missing for 8 (0.7%) on air pollution. Because variables included in regression models have a low proportion of missing data (3.8%), analyses were conducted as complete case.

### 2.5 Ethics Approval

This study was approved by the human subjects institutional review board (IRB) at UMass Amherst, and the parent trial was approved by the IRBs for all study sites and a data coordinating centre. All participants provided written informed consent. A data and safety monitoring board provided external oversight for the parent trial.

## 3 Results

Among 1223 IUI cycles observed during the FAZST trial (*n* = 592 couples) from Salt Lake City, there were 133 (10.9%) hCG+ pregnancies. Of those who became pregnant, there were 46 pregnancy losses (34.6%) and 87 live births (65.4%). As compared to the overall cohort from Salt Lake City, male IUI participants tended to be older (mean age 32.8 [SD 5.6] vs. 32.3 [SD 5.8] years), have a > $100,000 household income (25.7% vs. 21.5%), be married (97.3% vs. 95.2%), have a master's degree or higher (19.4% vs. 17.2%), and identify as non‐Hispanic White (83.1% vs. 82.0%) or Asian (5.1% vs. 3.4%) (Table 1). Compared to the overall cohort, female partners in the IUI group tended to be older (mean age 31.0 [SD 5.0] vs. 30.5 [SD 5.1] years) and have a lower body mass index (mean 27.8 [SD 7.4] vs. 28.4 [SD 8.1] kg/m^2^). Among the IUI group, a prior diagnosis of male‐factor fertility was more common (22.8% vs. 21.4%) and female‐factor infertility less common (34.4% vs. 36.0%) as compared to the overall cohort. For prior male‐factor infertility diagnosis, 58 reported low sperm count, 52 abnormal sperm morphology, 52 varicocele, 50 low sperm motility, 16 inguinal hernia repair, 12 hydrocele, 10 testicular injury, and 10 vasectomy reversal. For prior female‐factor infertility diagnosis, 68 reported polycystic ovary syndrome, 35 anovulation, 34 endometriosis, 16 diminished ovarian reserve, 8 blocked fallopian tubes, 8 uterine fibroids, and 5 uterine abnormalities. A total of 76 (12.8%) couples had one or more prior IUI attempts. At baseline, 282 (47.6%) had a semen quality parameter below the WHO 5th percentile.

**TABLE 1:** Baseline characteristics of FAZST participants in the Salt Lake City region.

Characteristic	Overall (*n* = 2015)	IUI cohort (*n* = 592 couples)
	*n* (%)	*n* (%)
Male partner age (years, mean [SD])	32.3 (5.8)	32.8 (5.6)
Female partner age (years, mean [SD])	30.5 (5.1)	31.0 (5.0)
Male partner BMI (kg/m^2^, mean [SD])	29.7 (6.7)	29.8 (6.7)
Female partner BMI (kg/m^2^, mean [SD])	28.4 (8.1)	27.8 (7.4)
Married		
Yes	1916 (95.2)	574 (97.3)
No	97 (4.8)	16 (2.7)
Male partner's highest degree		
Master's degree or higher	346 (17.2)	115 (19.4)
Bachelor's degree	611 (30.3)	187 (31.6)
Some college	738 (36.6)	212 (35.8)
High school or less	313 (15.5)	75 (12.7)
Did not wish to provide	7 (0.4)	3 (0.5)
Male partner's race/ethnicity		
Non‐Hispanic White	1653 (82.0)	492 (83.1)
Hispanic or Latino	121 (6.0)	29 (4.9)
Asian	69 (3.4)	30 (5.1)
Black or African American	31 (1.5)	10 (1.7)
Other race/ethnicity or do not wish to provide	141 (7.0)	27 (4.6)
Annual household income		
< $40,000	303 (15.7)	66 (11.5)
$40,000–< $75,000	791 (41.0)	227 (39.7)
$75,000–< $100,000	420 (21.8)	132 (23.1)
≥ $100,000	416 (21.5)	147 (25.7)
Self‐reported male infertility		
Yes	325 (21.4)	109 (22.8)
No	1192 (78.6)	369 (77.2)
Self‐reported female infertility		
Yes	546 (36.0)	164 (34.4)
No	969 (64.0)	313 (65.6)

*Note:* In the overall cohort, data missing for male partner body mass index (BMI, *n* = 2), female partner BMI (*n* = 8), being married (*n* = 2), income (*n* = 85), self‐reported male infertility (*n* = 498), and self‐reported female infertility (*n* = 500). In the IUI cohort, data is missing for female partner BMI (*n* = 2), being married (*n* = 3), income (*n* = 20), self‐reported male infertility (*n* = 114), and self‐reported female infertility (*n* = 115).

Median ambient air pollution for the 74‐day spermatogenesis window leading up to the first IUI cycle was 6.5 (IQR 5.0, 8.7) ug/m^3^ for PM_2.5_, 8.1 (IQR 5.4, 12.3) ppb for NO_2_, 34.5 (IQR 25.3, 40.8) ppb for O_3_, and 0.38 (IQR 0.25, 0.62) ppb for SO_2_ (Table 2). Air pollutants were strongly positively correlated with each other, except for O_3_, which was negatively correlated with all other pollutants (Figure S3). Correlations were stronger in the cold versus warm season between PM_2.5_ and NO_2_ (*r* = 0.78 vs. *r* = 0.28) and PM_2.5_ and O_3_ (*r* = −0.71 vs. *r* = −0.05) (Figures S4 and S5). Correlation coefficients ranged between 0.21 and 0.63 across windows of spermatogenesis for PM_2.5_, 0.68 and 0.92 for NO_2_, 0.49 and 0.91 for O_3_, and 0.75 and 0.87 for SO_2_ (Table S1).

**TABLE 2:** Distribution of air pollutants during the 74‐day spermatogenesis window at the first cycle of follow‐up (*n* = 592).

Air pollutant	Overall			Warm season (April–September)	Cold season (October–March)
	Minimum	Maximum	Median (IQR)	Median (IQR)	Median (IQR)
PM_2.5_ (μg/m^3^)	1.3	24.2	6.5 (5.0, 8.7)	5.7 (4.7, 7.0)	7.7 (5.7, 10.4)
NO_2_ (ppb)	0.24	33.6	8.1 (5.4, 12.3)	5.9 (4.1, 8.1)	12.1 (8.3, 17.1)
O_3_ (ppb)	10.3	50.1	34.5 (25.3, 40.8)	40.5 (36.7, 42.8)	25.2 (19.6, 30.4)
SO_2_ (ppb)	0.08	3.5	0.38 (0.25, 0.62)	0.31 (0.21, 0.52)	0.44 (0.31, 0.67)

Abbreviations: NO_2_, nitrogen dioxide; O_3_, ozone; PM_2.5_, particulate matter < 2.5 μm; SO_2_, sulfur dioxide.

Overall, we observed an association between greater exposure to O_3_ and NO_2_ during spermatogenesis and a lower likelihood of pregnancy and live birth in multipollutant and mixture models, with fewer clear associations for pregnancy loss. In single‐pollutant models, O_3_ during spermatogenesis was associated with a 16% lower chance of pregnancy (RR 0.84, 95% CI 0.73, 0.97), while PM_2.5_ and NO_2_ were associated with a higher chance of pregnancy (RR 1.27, 95% CI 0.98, 1.65 and RR 1.13, 95% CI 0.95, 1.33), although estimates were less precise (Table 3). In multipollutant models, O_3_ was associated with a 29% lower chance of pregnancy (95% CI 0.55, 0.93) and NO_2_ with a 27% lower chance of pregnancy (95% CI 0.50, 1.05), while PM_2.5_ remained associated with a higher chance of pregnancy (RR 1.31, 95% CI 0.92, 1.85). Findings from the mixture model were similar to those from the multipollutant model, with O_3_ and NO_2_ contributing negatively (lower chance of pregnancy) and PM_2.5_ contributing positively to the mixture (Table S2). A decile increase in the air pollution mixture was associated with a 21% lower chance of pregnancy (95% CI 0.62, 1.01).

**TABLE 3:** Average exposure to air pollutants during the 74‐day window of spermatogenesis and hCG+ pregnancy, pregnancy loss, and live birth in intrauterine insemination (IUI) cycles (*n* = 1176).^a^

	hCG+ pregnancy	Pregnancy loss^b^	Live birth
	RR (95% CI)	RR (95% CI)	RR (95% CI)
Single‐pollutant model			
PM_2.5_ (per 5 μg/m^3^)	1.27 (0.98, 1.65)	1.02 (0.71, 1.46)	1.26 (0.91, 1.75)
NO_2_ (per 5 ppb)	1.13 (0.95, 1.33)	0.98 (0.77, 1.23)	1.15 (0.94, 1.41)
O_3_ (per 5 ppb)	0.84 (0.73, 0.97)	0.99 (0.81, 1.20)	0.84 (0.71, 1.00)
SO_2_ (per 0.5 ppb)	0.97 (0.82, 1.14)	0.99 (0.80, 1.23)	0.99 (0.81, 1.21)
Multipollutant model			
PM_2.5_ (per 5 μg/m^3^)	1.31 (0.92, 1.85)	0.98 (0.53, 1.80)	1.22 (0.79, 1.89)
NO_2_ (per 5 ppb)	0.73 (0.50, 1.05)	0.89 (0.49, 1.60)	0.82 (0.52, 1.29)
O_3_ (per 5 ppb)	0.71 (0.55, 0.93)	0.90 (0.61, 1.35)	0.76 (0.55, 1.06)
SO_2_ (per 0.5 ppb)	0.95 (0.80, 1.14)	1.03 (0.80, 1.32)	0.96 (0.77, 1.20)
Air pollution mixture (per decile increase)	0.79 (0.62, 1.01)	0.97 (0.64, 1.48)	0.81 (0.60, 1.09)

Abbreviations: NO_2_, nitrogen dioxide; O_3_, ozone; PM_2.5_, particulate matter < 2.5 μm; SO_2_, sulfur dioxide.

^a^ All models adjust for male age, season, and income. Multipollutant and mixture models additionally adjust for co‐pollutants PM_2.5_, NO_2_, O_3_, and SO_2_.

^b^ Inverse probability weighting accounted for factors associated with pregnancy.

O_3_ and NO_2_ were most strongly associated with a lower chance of pregnancy early in spermatogenesis during mitosis (RR 0.77, 95% CI 0.64, 0.93 and RR 0.70, 95% CI 0.51, 0.96, respectively) and meiosis I + II (RR 0.76, 95% CI 0.61, 0.96 and RR 0.80, 95% CI 0.58, 1.11, respectively) in multipollutant models (Table S2, Figure 3). In mixture models, associations of a decile increase in the mixture with lower chance of pregnancy were strongest during mitosis (RR 0.83, 95% CI 0.70, 0.99), meiosis I + II (RR 0.84, 95% CI 0.67, 1.06), and spermiation (RR 0.81, 95% CI 0.68, 0.96) (Table S3, Figure 3), with both O_3_ and NO_2_ contributing to a lower chance of pregnancy in the mixture at each timepoint (Figure S6). We observed a trend of higher O_3_, NO_2_, and SO_2_ with lower chance of pregnancy during the days leading up to ovulation and the approximate window of implantation in multipollutant models, and the association of a decile increase in the air pollution mixture with lower chance of pregnancy leading up to ovulation (RR 0.90, 95% CI 0.77, 1.06) and during implantation (RR 0.88, 95% CI 0.73, 1.06) (Table S3, Figure S7).

**FIGURE 3:**
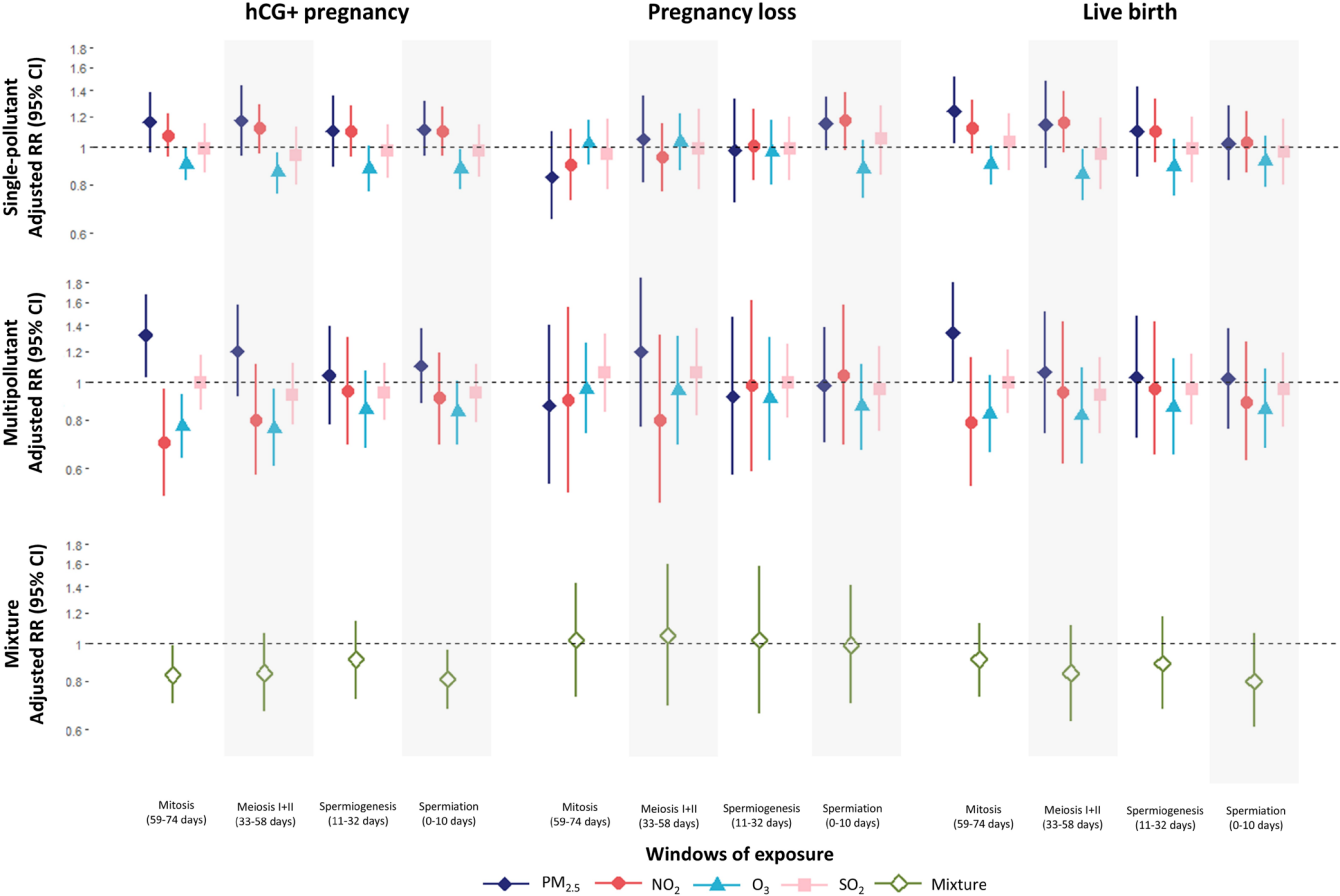
Relationship between exposure to ambient air pollutants across windows of susceptibility during spermatogenesis and outcomes of intrauterine insemination cycles for single‐pollutant, multipollutant, and mixture models (*n* = 1176). Particulate matter < 2.5 μm (dark blue, PM_2.5_) modelled per 5 μg/m^3^, nitrogen dioxide (red, NO_2_) and ozone (light blue, O_3_) modelled per 5 ppb, sulfur dioxide (pink, SO_2_) modelled per 0.5 ppb, and air pollution mixture (green) modelled per decile increase. All models adjusted for male partner age, season, and income, and multipollutant models jointly adjust for PM_2.5_, NO_2_, O_3_, and SO_2_. Inverse probability weighting was used in pregnancy loss models to account for pregnancy‐related factors.

Findings for live birth were similar to findings for pregnancy, although estimates were less precise. O_3_ during spermatogenesis and all spermatogenesis developmental windows was associated with a trend of lower probability of live birth (e.g., RR 0.76, 95% CI 0.55, 1.06 for spermatogenesis) (Table S4). NO_2_ was similarly associated with a lower chance of live birth during spermatogenesis, whereas PM_2.5_ during mitosis was associated with an increased chance of live birth in both single‐ and multipollutant models. We also observed a lower probability of live birth with O_3_ during implantation (RR 0.81, 95% CI 0.66, 0.99) (Table S4, Figure S8).

Overall, no clear patterns emerged between air pollutant exposure and pregnancy loss (Table S5, Figure 3). However, PM_2.5_ exposure during the post‐implantation window was associated with a higher risk of pregnancy loss in multipollutant (RR 1.21, 95% CI 1.08, 1.36) and single pollutant (RR 1.19, 95% CI 1.07, 1.32) models (Table S5).

In secondary analyses, we observed similar findings when adjusting for female partner age (Table S6), prior male‐factor infertility diagnosis (Table S7), and baseline semen quality (Table S8). We also observed similar findings when restricting analyses to the first IUI cycle of follow‐up (Table S9) and when mutually adjusting for windows across spermatogenesis (Table S10). No clear differences were observed by treatment assignment (Tables S11 and S12), prior male‐factor infertility diagnosis (Tables S13 and S14), or baseline semen quality (Tables S15 and S16) for hCG+ pregnancy or livebirth. O_3_ and NO_2_ during spermatogenesis, mitosis, and meiosis I + II in the cold season were related to a trend of lower probability of IUI pregnancy (e.g., RR 0.61, 95% CI 0.41, 0.90 and RR 0.63, 95% CI 0.40, 1.00, respectively, for spermatogenesis) (Figure 4, Table S2), although wider confidence intervals for RERI limited clear inferences for additive interactions. The air pollution mixture during spermatogenesis, mitosis, and meiosis I + II in the cold season was also related to a trend of lower probability of pregnancy and live birth (e.g., RR 0.57, 95% CI 0.38, 0.86 and RR 0.58, 95% CI 0.35, 0.97, respectively, for spermatogenesis) (Figure 4, Table S2).

**FIGURE 4:**
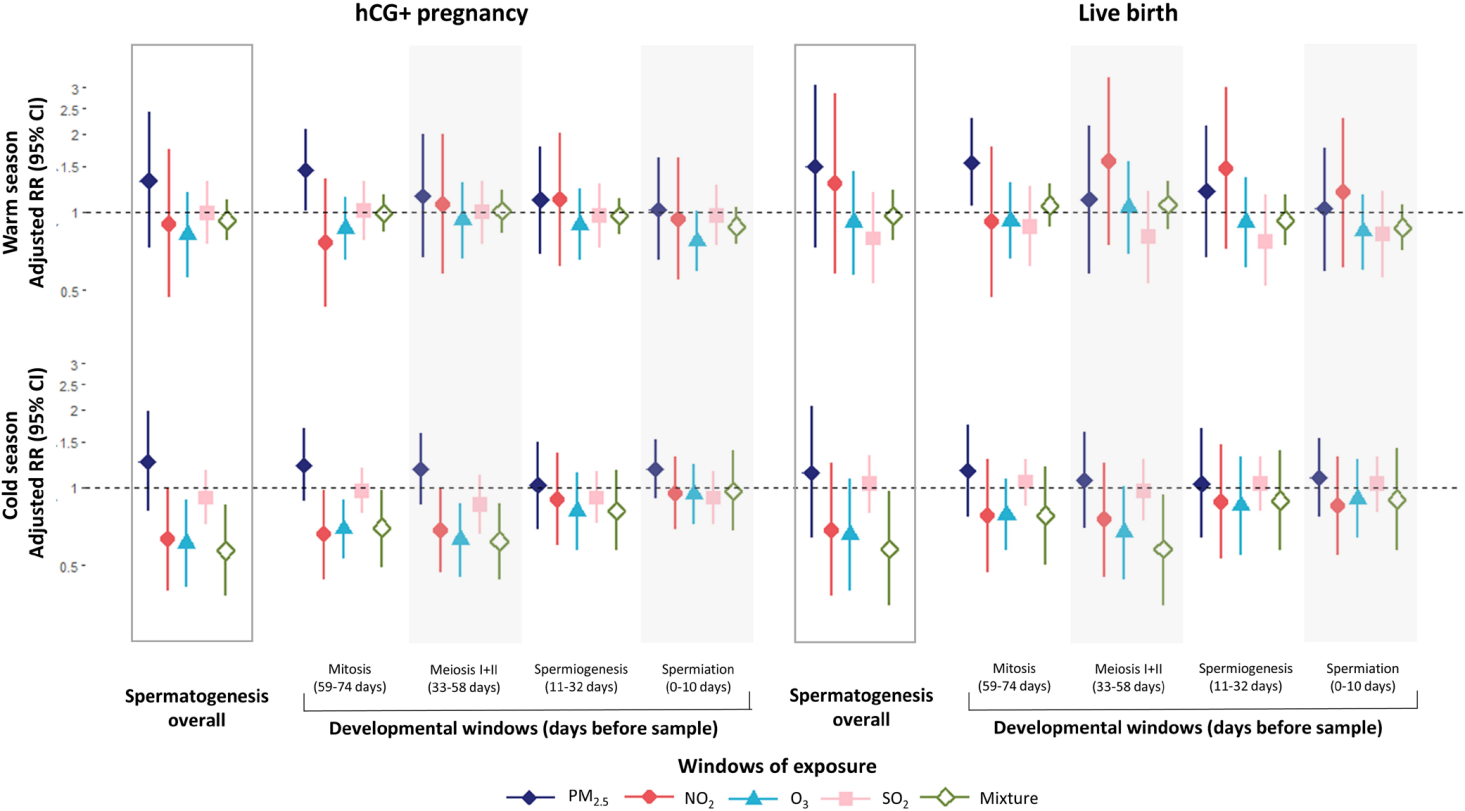
Relationship between exposure to ambient air pollutants across spermatogenesis and windows of susceptibility during spermatogenesis and outcomes of intrauterine insemination cycles for multipollutant and mixture models by warm (April–September) versus cold (October–March) season (*n* = 1176). Particulate matter < 2.5 μm (dark blue, PM_2.5_) modelled per 5 μg/m^3^, nitrogen dioxide (red, NO_2_) and ozone (light blue, O_3_) modelled per 5 ppb, sulfur dioxide (pink, SO_2_) modelled per 0.5 ppb, and mixture (green) modelled per decile increase. All models adjusted for male partner age, season, and income, and multipollutant models jointly adjust for PM_2.5_, NO_2_, O_3_, and SO_2_. Inverse probability weighting was used in pregnancy loss models to account for pregnancy‐related factors.

## 4 Comment

### 4.1 Principal Findings

Our findings suggest exposure to O_3_ and NO_2_ during spermatogenesis may be associated with a lower probability of hCG+ pregnancy and live birth among couples receiving IUI treatment. While exposure to O_3_ and NO_2_ throughout spermatogenesis was associated with a lower probability of hCG+ pregnancy and live birth, findings suggest that exposure during the developmental windows of mitosis and meiosis I + II may be particularly important. As no clear patterns emerged for air pollutants and pregnancy loss, findings may suggest that impacts on hCG+ pregnancy play the largest role in observed associations between paternal air pollution exposure and live birth [35]. Differences between single‐pollutant versus multipollutant and mixture models suggest co‐pollutant confounding was important in this study population residing in the Salt Lake region and emphasise the importance of disentangling pollution mixtures.

### 4.2 Strengths of the Study

Strengths of this study include consideration of both male and female partner exposure to ambient air pollution and a focus on couples receiving IUI treatment. Windows of susceptibility to air pollution were determined a priori based on biological estimates of the duration of spermatogenesis stages [28]. We implemented several modelling approaches to identify air pollution effects, including multipollutant models and mixture models to account for pollutant interdependencies. We also minimised bias by incorporating multivariable models that accounted for area‐level and seasonal confounders, and IPWs to address potential selection bias in models for pregnancy loss.

### 4.3 Limitations of the Data

A key limitation of our analyses is the inability to tease out partner‐specific effects prior to the insemination date. Our investigation of biologically relevant developmental windows across spermatogenesis provides some basis for interpretation, but findings require further research to better disentangle partner‐specific effects. Additionally, the identified developmental windows of exposure are estimates, and due to uncertainty in window approximations, we cannot say with strong assurance whether mitosis and meiosis I‐II are truly critical periods of exposure. Salt Lake City air quality is influenced by a complex mixture of pollutants, non‐linear chemistry, and large‐scale meteorology that can lead to co‐pollutant confounding [36]. We addressed co‐pollutant confounding by incorporating multipollutant and mixture models, although we could not include all relevant pollutants, and residual confounding is likely. Additionally, we could not separate personal or outdoor ambient air pollution exposure, and the 12 km × 12 km resolution of the CMAQ model may have led to misclassification of exposure and potentially attenuated findings [37, 38]. Finally, because study participants were largely non‐Hispanic White and had moderate to high incomes, the results of this study should be generalised with caution [39]. Findings will benefit from replication in more diverse cohorts and in areas with varying exposures to air pollution mixtures.

### 4.4 Interpretation

Men's exposure to ambient air pollution may have downstream impacts on infertility treatment through adverse effects on semen quality [40], increased DNA strand breaks [41], and epigenetic changes in sperm. We observed the strongest impacts of air pollution on IUI outcomes with exposure during the cell division stages of mitosis and meiosis I + II in spermatogenesis, during which haploid spermatids are formed, and DNA methylation signatures are finalised [42], setting the stage for later sperm morphological development and fertilisation capacity. Early spermatogenesis may be a particularly sensitive window for the effects of air pollution on semen quality, including reduced motility [43, 44, 45] and fewer morphologically normal sperm [9]. In the FASZT trial, we previously observed that higher O_3_ during meiosis I + II and spermiogenesis was associated with lower sperm morphology [46].

Similar to research in both fecund and IVF treatment populations [1, 2, 3, 6], we observed associations between air pollution leading up to ovulation induction and during implantation and hCG+ pregnancy. However, observations of similarly strong associations within the estimated windows of mitosis and meiosis I + II suggest that reframing partner‐specific windows of vulnerability may be important for understanding preconception environmental exposures and couple‐level outcomes. This study expands on prior research in IVF and natural pregnancy planning populations by focusing on outcomes of IUI cycles, for which male‐partner effects of air pollution may be more similar to natural pregnancy planning populations [47]. Because IUI is often a first‐line treatment for unexplained infertility and has considerably fewer side effects and is lower cost than IVF [26], understanding modifiable factors that influence outcomes of IUI is critical for helping couples achieve a live birth with the minimal interventions necessary.

## 5 Conclusions

Findings suggest exposure to O_3_ and NO_2_ during early spermatogenesis, along with exposure during ovulation and implantation, may be related to a lower probability of hCG+ pregnancy and live birth among couples undergoing IUI treatment. We observed less evidence of an association between PM_2.5_ and SO_2_ and IUI outcomes. While the findings are suggestive, more data are needed to distinguish partner effects, including incorporating personal exposure to air pollution and assessing mediation by biomarkers of sperm function. Men's preconception health remains an understudied area, and future work is needed to better identify windows of susceptibility and pathways of effect for air pollution and outcomes of infertility treatment.

## Author Contributions

C.N. and J.R.P. were responsible for the design of the study. T.P.C., A.S. and A.M.R. were responsible for estimation of air quality and meteorological data and provided advising on modelling and interpretation of exposure data. K.C.S., M.S. and R.B.H. were responsible for geocoding and linkage of air quality and meteorological data and K.C.S. and J.A.V. provided critical feedback on regional considerations for modelling and interpretation of findings. C.M.P. and E.B.J. provided input on clinical interpretation of findings and, along with L.M.R., C.N. and K.R., contributed to data cleaning for clinical endpoints. L.M.R. and C.N. were responsible for statistical analysis, and N.J.P., P.M. and J.R.P. advised on statistical methods. J.R.P., T.P.C., N.J.P., P.M., K.C.S., J.A.V., C.M.P., E.B.J. and K.R. provided critical feedback on the interpretation of results. L.M.R. drafted the manuscript, and all authors contributed to manuscript revisions.

## Funding

This ancillary study was supported by funding from the National Institute of Environmental Health Sciences (K01‐ES034005). The parent trial was supported by funding from the Intramural Research Program of the *Eunice Kennedy Shriver* National Institute of Child Health and Human Development (HHSN275201200007C and HHSN275201300026).

## Conflicts of Interest

The authors declare no conflicts of interest.

## Supporting information


**Figure S1:** Flowchart for inclusion/exclusion for participants and IUI cycles from original FAZST cohort.
**Figure S2:** Windows of susceptibility and developmental stages during spermatogenesis.
**Figure S3:** Pearson Correlation Plot: Criteria Air Pollutants (Overall, Jan‐Dec) 74‐Day Spermatogenesis Window (*n* = 588 couples). PM_2.5_: particulate matter < 2.5 μm; NO_2_: nitrogen dioxide; O_3_: ozone: SO_2_: sulfur dioxide.
**Figure S4:** Pearson Correlation Plot: Criteria Air Pollutants 74‐Day Spermatogenesis Window (Warm Season, April‐September) (*n* = 305 couples). PM_2.5_: particulate matter < 2.5 μm; NO_2_: nitrogen dioxide; O_3_: ozone: SO_2_: sulfur dioxide.
**Figure S5:** Pearson Correlation Plot: Criteria Air Pollutants, 74‐Day Spermatogenesis Window (Cold Season, October–March) (*n* = 283 couples). PM_2.5_: particulate matter < 2.5 μm; NO_2_: nitrogen dioxide; O_3_: ozone: SO_2_: sulfur dioxide.
**Figure S6:** Relative weights for each air pollutant in quantile g‐computation models by window of exposure and outcome. PM_2.5_: Particulate matter < 2.5 μm; NO_2_: Nitrogen dioxide; O_3_: Ozone; SO_2_: Sulfur dioxide (*n* = 1176 for pregnancy and live birth and *n* = 127 for pregnancy loss).
**Figure S7:** Relationship between exposure to ambient air pollutants across windows of susceptibility during ovulation and implantation and outcomes of intrauterine insemination cycles for single‐pollutant, multipollutant, and mixture models (*n* = 1176 for pregnancy and live birth and *n* = 127 for pregnancy loss). Particulate matter < 2.5 μm (dark blue, PM_2.5_) modelled per 5 μg/m^3^, nitrogen dioxide (red, NO_2_) and ozone (light blue, O_3_) modelled per 5 ppb, sulfur dioxide (pink, SO_2_) modelled per 0.5 ppb, and mixture (green) modelled per decile increase. All models adjust for men's age, season, and household income, and multipollutant models jointly adjust for PM_2.5_, NO_2_, O_3_, and SO_2_.
**Figure S8:** Relationship between exposure to ambient air pollutants across windows of susceptibility during ovulation and implantation and outcomes of intrauterine insemination cycles for multipollutant, and mixture models by warm (April 1–September 30) versus cold (October 1–March 31) season (*n* = 1176). Particulate matter < 2.5 μm (dark blue, PM_2.5_) modelled per 5 μg/m^3^, nitrogen dioxide (red, NO_2_) and ozone (light blue, O_3_) modelled per 5 ppb, sulfur dioxide (pink, SO_2_) modelled per 0.5 ppb, and mixture (black) modelled per decile increase. All models adjusted for men's age, season, and household income, and multipollutant models jointly adjust for PM_2.5_, NO_2_, O_3_, and SO_2_.
**Table S1:** Spearman correlation coefficients across windows of spermatogenesis by air pollutant (*n* = 1176).
**Table S2:** Association of air pollution during spermatogenesis, ovulation, implantation, and pregnancy in intrauterine insemination (IUI) cycles: multipollutant model, single‐pollutant model, and effect modification by season (multipollutant) (*n* = 1176).
**Table S3:** Association of decile change in the air pollution mixture and hCG+ pregnancy, pregnancy loss, and live birth in intrauterine insemination (IUI) cycles (*n* = 1176).
**Table S4:** Association of air pollution during spermatogenesis, ovulation, implantation, and live birth in intrauterine insemination (IUI) cycles: multipollutant model, single‐pollutant model, and effect modification by season (multipollutant) (*n* = 1176).
**Table S5:** Association of air pollution during spermatogenesis, ovulation, implantation, and pregnancy loss^a^ in intrauterine insemination (IUI) cycles: multipollutant and single‐pollutant models (among *n* = 127 with an observed pregnancy).
**Table S6:** Association of air pollution with pregnancy, pregnancy loss^a^, and live birth in intrauterine insemination (IUI) cycles additionally adjusted for female partner age: multipollutant and single‐pollutant models (*n* = 1176).
**Table S7:** Association of air pollution with pregnancy, pregnancy loss^a^, and live birth in intrauterine insemination (IUI) cycles additionally adjusted for self‐reported prior male‐factor infertility diagnosis: multipollutant and single‐pollutant models (*n* = 1176).
**Table S8:** Association of air pollution with pregnancy, pregnancy loss^a^, and live birth in intrauterine insemination (IUI) cycles additionally adjusted for a baseline semen quality parameter below WHO 5% percentile: multipollutant and single‐pollutant models.
**Table S9:** Association of air pollution with pregnancy, pregnancy loss^a^, and live birth in intrauterine insemination (IUI) cycles restricted to first IUI cycle of follow‐up: multipollutant and single‐pollutant models.
**Table S10:** Association of air pollution with pregnancy, pregnancy loss^a^, and live birth in intrauterine insemination (IUI) cycles additionally co‐adjusted for windows of exposure during spermatogenesis: multipollutant and single‐pollutant models.
**Table S11:** Association of air pollution and pregnancy in intrauterine insemination (IUI) cycles by treatment assignment (multipollutant models) (*n* = 1176).
**Table S12:** Association of air pollution and live birth in intrauterine insemination (IUI) cycles by treatment assignment (*n* = 1176).
**Table S13:** Association of air pollution and pregnancy in intrauterine insemination (IUI) cycles by history of male partner infertility diagnosis (*n* = 1176).
**Table S14:** Association of air pollution and live birth in intrauterine insemination (IUI) cycles by history of male partner infertility diagnosis (*n* = 1176).
**Table S15:** Association of air pollution and pregnancy in intrauterine insemination (IUI) cycles by baseline semen quality parameters < WHO 5th percentile thresholds (*n* = 1158).
**Table S16:** Association of air pollution and live birth in intrauterine insemination (IUI) cycles by baseline semen quality parameters < WHO 5th percentile thresholds (*n* = 1158).

## Data Availability

Consistent with NIH policy, data can be made available to other researchers. For details, please see http://grants.nih.gov/grants/policy/data_sharing/ for the NIH data sharing policy.
