# Supplementary material for: Critical Windows of Air Pollution Exposure During Spermatogenesis and Intrauterine Insemination Outcomes
Source: Paediatr Perinat Epidemiol. 2026 Aug 4;40(7):774–84. doi: 10.1111/ppe.70185 (PMC13624516; doi:10.1111/ppe.70185)
Supplement: Supplementary file 1 [file PPE-40-774-s001.docx]

**Supplemental Material**

Critical Windows of Air Pollution Exposure During Spermatogenesis and Intrauterine Insemination Outcomes

Lindsey M. Russo^1^, J. Richard Pilsner^2^, Timothy P. Canty^3^, Neil J. Perkins^4^, Pauline Mendola^5^, Karen C. Schliep^6^, May Shaaban^6^, Rachael B. Hemmert^6^, Akanksha Singh^3^, Allison M. Ring^3^, James A. Vanderslice^6^, C. Matthew Peterson^7^, Erica B. Johnstone^7^, Kaniz Rabeya^8^, Carrie Nobles^8^

^1^Division of General Internal Medicine, Weill Cornell Medicine, 420 East 70^th^ Street, New York, NY 10021, USA; ^2^Department of Obstetrics and Gynecology, Wayne State University School of Medicine, Detroit, MI, 48201, USA; ^3^Department of Atmospheric and Oceanic Science, The University of Maryland, College Park, MD, 20742, USA; ^4^Biostatistics and Bioinformatics Branch, *Eunice Kennedy Shriver* National Institute of Child Health and Human Development, National Institutes of Health, Bethesda, MD, 20817, USA; ^5^Epidemiology and Environmental Health, School of Public Health and Health Professions, University at Buffalo, Buffalo, NY, 14215, USA’; ^6^Division of Public Health, Department of Family and Preventive Medicine, University of Utah Health, Salt Lake City, Utah, USA; ^7^Division of Reproductive Endocrinology and Infertility, Department of Obstetrics and Gynecology, University of Utah Health, Salt Lake City, Utah, USA; ^8^Department of Environmental Health Sciences, School of Public Health and Health Sciences, University of Massachusetts Amherst, Amherst, Massachusetts, USA.

**Table of Contents:**

Table S1. Spearman correlation coefficients across windows of spermatogenesis by air pollutant.

Table S2. Association of air pollution during spermatogenesis, ovulation, implantation, and pregnancy in intrauterine insemination (IUI) cycles: multipollutant model, single-pollutant model, and effect modification by season (multipollutant) (n=1,176).

Table S3. Association of decile change in the air pollution mixture and hCG+ pregnancy, pregnancy loss, and live birth in intrauterine insemination (IUI) cycles (n=1,176).

Table S4. Association of air pollution during spermatogenesis, ovulation, implantation, and live birth in intrauterine insemination (IUI) cycles: multipollutant model, single-pollutant model, and effect modification by season (multipollutant) (n=1,176).

Table S5. Association of air pollution during spermatogenesis, ovulation, implantation, and pregnancy loss^a^ in intrauterine insemination (IUI) cycles: multipollutant and single-pollutant models (among n=127 with an observed pregnancy).

Table S6. Association of air pollution with pregnancy, pregnancy loss^a^, and live birth in intrauterine insemination (IUI) cycles additionally adjusted for female partner age: multipollutant and single-pollutant models.

Table S7. Association of air pollution with pregnancy, pregnancy loss^a^, and live birth in intrauterine insemination (IUI) cycles additionally adjusted for male partner prior infertility diagnosis: multipollutant and single-pollutant models.

Table S8. Association of air pollution with pregnancy, pregnancy loss^a^, and live birth in intrauterine insemination (IUI) cycles additionally adjusted for baseline semen quality parameters below WHO 5% thresholds: multipollutant and single-pollutant models.

Table S9. Association of air pollution with pregnancy, pregnancy loss^a^, and live birth in intrauterine insemination (IUI) cycles restricted to first IUI cycle of follow-up: multipollutant and single-pollutant models

Table S10. Association of air pollution with pregnancy, pregnancy loss^a^, and live birth in intrauterine insemination (IUI) cycles additionally co-adjusted for windows of exposure during spermatogenesis: multipollutant and single-pollutant models.

Table S11. Association of air pollution and pregnancy in intrauterine insemination (IUI) cycles by treatment assignment (multipollutant models) (n=1,176).

Table S12. Association of air pollution and live birth in intrauterine insemination (IUI) cycles by treatment assignment (n=1,176).

Table S13. Association of air pollution and pregnancy in intrauterine insemination (IUI) cycles by history of male partner infertility diagnosis (n=1,176).

Table S14. Association of air pollution and live birth in intrauterine insemination (IUI) cycles by history of male partner infertility diagnosis (n=1,176).

Table S15. Association of air pollution and pregnancy in intrauterine insemination (IUI) cycles by baseline semen quality parameters <WHO 5^th^ percentile thresholds (n=1,176).

Table S16. Association of air pollution and live birth in intrauterine insemination (IUI) cycles by baseline semen quality parameters <WHO 5^th^ percentile thresholds (n=1,176).

Figure S1. Flowchart for inclusion/exclusion for participants and IUI cycles from original FAZST cohort.

Figure S2. Windows of susceptibility and developmental stages during spermatogenesis

Figure S3. Pearson Correlation Plot: Criteria Air Pollutants (Overall, Jan-Dec) 74-Day Spermatogenesis Window *(n*=501 couples).

Figure S4. Pearson Correlation Plot: Criteria Air Pollutants 74-Day Spermatogenesis Window (Warm Season, Apr-Sept) (n=241 couples).

Figure S5. Pearson Correlation Plot: Criteria Air Pollutants, 74-Day Spermatogenesis Window (Cold Season, Oct-March) *(n*=260 couples).

Figure S6. Relative weights for each air pollutant in quantile g-computation models by window of exposure and outcome

Figure S7. Relationship between exposure to ambient air pollutants across windows of susceptibility during ovulation and implantation and outcomes of intrauterine insemination cycles for single-pollutant, multipollutant, and mixture models.

Figure S8. Relationship between exposure to ambient air pollutants across windows of susceptibility during ovulation and implantation and outcomes of intrauterine insemination cycles for multipollutant, and mixture models by warm (Apr. 1 – Sept. 30) versus cold (Oct.1 – Mar. 31) season.

Table S1. Spearman correlation coefficients across windows of spermatogenesis by air pollutant (n=1,176).

|  | Mitosis | Meiosis I+II | Spermiogenesis | Spermiation |
| --- | --- | --- | --- | --- |
| **Particulate matter <2.5 microns** |  |  |  |  |
| Mitosis (59-74 days before sample) | 1 | 0.62 | 0.36 | 0.21 |
| Meiosis I+II (33-58 days before sample) |  | 1 | 0.61 | 0.41 |
| Spermiogenesis (11-32 days before sample) |  |  | 1 | 0.63 |
| Spermiation (0-10 days before sample) |  |  |  | 1 |
| **Nitrogen dioxide** |  |  |  |  |
| Mitosis (59-74 days before sample) | 1 | 0.92 | 0.80 | 0.68 |
| Meiosis I+II (33-58 days before sample) |  | 1 | 0.91 | 0.82 |
| Spermiogenesis (11-32 days before sample) |  |  | 1 | 0.92 |
| Spermiation (0-10 days before sample) |  |  |  | 1 |
| **Ozone** |  |  |  |  |
| Mitosis (59-74 days before sample) | 1 | 0.90 | 0.69 | 0.49 |
| Meiosis I+II (33-58 days before sample) |  | 1 | 0.89 | 0.74 |
| Spermiogenesis (11-32 days before sample) |  |  | 1 | 0.91 |
| Spermiation (0-10 days before sample) |  |  |  | 1 |
| **Sulfur dioxide** |  |  |  |  |
| Mitosis (59-74 days before sample) | 1 | 0.86 | 0.82 | 0.75 |
| Meiosis I+II (33-58 days before sample) |  | 1 | 0.88 | 0.82 |
| Spermiogenesis (11-32 days before sample) |  |  | 1 | 0.87 |
| Spermiation (0-10 days before sample) |  |  |  | 1 |

Table S2. Association of air pollution during spermatogenesis, ovulation, implantation, and pregnancy in intrauterine insemination (IUI) cycles: multipollutant model, single-pollutant model, and effect modification by season (multipollutant) (n=1,176).

|  | | Pregnancy | | | | | |
| --- | --- | --- | --- | --- | --- | --- | --- |
|  | | Overall | | By season | | | |
|  | | Multipollutant | Single-pollutant | Multipollutant | | | |
|  | |  |  | Warm season | | Cold season | RERI |
|  | | RR (95% CI) | RR (95% CI) | RR (95% CI) | | RR (95% CI) | Estimate (95% CI) |
| Spermatogenesis (0-74 days before sample) | | |  |  | |  |  |
| PM_2.5_ (5 μg/m^3^) | | 1.31 (0.92, 1.85) | 1.27 (0.98, 1.65) | 1.32 (0.73, 2.41) | | 1.26 (0.81, 1.97) | 0.064 (-0.035, 0.199) |
| NO_2_ (5 ppb) | | 0.73 (0.50, 1.05) | 1.13 (0.95, 1.33) | 0.90 (0.47, 1.74) | | 0.63 (0.40, 1.00) | -0.027 (-0.205, 0.086) |
| O_3_ (5 ppb) | | 0.71 (0.55, 0.93) | 0.84 (0.73, 0.97) | 0.82 (0.56, 1.19) | | 0.61 (0.41, 0.90) | -0.020 (-0.144, 0.061) |
| SO_2_ (0.5 ppb) | | 0.95 (0.80, 1.14) | 0.97 (0.82, 1.14) | 0.99 (0.75, 1.32) | | 0.91 (0.72, 1.16) | 0.016 (-0.017, 0.064) |
| Mitosis (59-74 days before sample) | | |  |  | |  |  |
| PM_2.5_ (5 μg/m^3^) | | 1.32 (1.03, 1.68) | 1.16 (0.97, 1.38) | 1.45 (1.01, 2.08) | | 1.22 (0.89, 1.69) | 0.044 (-0.032, 0.144) |
| NO_2_ (5 ppb) | | 0.70 (0.51, 0.96) | 1.07 (0.94, 1.22) | 0.76 (0.43, 1.34) | | 0.66 (0.44, 0.98) | -0.023 (-0.147, 0.070) |
| O_3_ (5 ppb) | | 0.77 (0.64, 0.93) | 0.90 (0.82, 1.00) | 0.86 (0.65, 1.14) | | 0.69 (0.53, 0.90) | -0.003 (-0.070, 0.052) |
| SO_2_ (0.5 ppb) | | 1.00 (0.85, 1.17) | 0.99 (0.86, 1.15) | 1.01 (0.78, 1.31) | | 0.97 (0.79, 1.19) | 0.012 (-0.015, 0.047) |
| Meiosis I+II (33-58 days before sample) | | |  |  | |  |  |
| PM_2.5_ (5 μg/m^3^) | | 1.20 (0.92, 1.58) | 1.17 (0.95, 1.44) | 1.15 (0.67, 1.98) | | 1.18 (0.86, 1.62) | 0.023 (-0.061, 0.128) |
| NO_2_ (5 ppb) | | 0.80 (0.58, 1.11) | 1.12 (0.96, 1.29) | 1.07 (0.58, 1.98) | | 0.68 (0.47, 1.00) | 0.000 (-0.126, 0.082) |
| O_3_ (5 ppb) | | 0.76 (0.61, 0.96) | 0.86 (0.76, 0.97) | 0.93 (0.66, 1.30) | | 0.63 (0.45, 0.87) | 0.003 (-0.071, 0.064) |
| SO_2_ (0.5 ppb) | | 0.93 (0.78, 1.12) | 0.95 (0.80, 1.13) | 1.00 (0.75, 1.32) | | 0.86 (0.66, 1.12) | 0.024 (-0.006, 0.063) |
| Spermiogenesis (11-32 days before sample) | | |  |  | |  |  |
| PM_2.5_ (5 μg/m^3^) | | 1.04 (0.78, 1.39) | 1.10 (0.89, 1.36) | 1.11 (0.69, 1.78) | | 1.02 (0.69, 1.50) | 0.058 (-0.010, 0.137) |
| NO_2_ (5 ppb) | | 0.95 (0.69, 1.30) | 1.10 (0.94, 1.28) | 1.12 (0.62, 2.02) | | 0.90 (0.60, 1.36) | -0.015 (-0.119, 0.065) |
| O_3_ (5 ppb) | | 0.85 (0.68, 1.07) | 0.88 (0.77, 1.01) | 0.89 (0.65, 1.23) | | 0.81 (0.57, 1.14) | -0.021 (-0.092, 0.037) |
| SO_2_ (0.5 ppb) | | 0.94 (0.80, 1.12) | 0.98 (0.83, 1.14) | 0.97 (0.73, 1.28) | | 0.91 (0.73, 1.15) | 0.009 (-0.024, 0.044) |
| Spermiation (0-10 days before sample) | | |  |  | |  |  |
| PM_2.5_ (5 μg/m^3^) | | 1.10 (0.88, 1.37) | 1.11 (0.95, 1.31) | 1.02 (0.65, 1.61) | | 1.18 (0.91, 1.53) | 0.014 (-0.048, 0.077) |
| NO_2_ (5 ppb) | | 0.91 (0.69, 1.19) | 1.10 (0.95, 1.27) | 0.94 (0.55, 1.61) | | 0.95 (0.69, 1.32) | -0.032 (-0.127, 0.051) |
| O_3_ (5 ppb) | | 0.84 (0.69, 1.01) | 0.88 (0.78, 0.99) | 0.77 (0.59, 1.01) | | 0.94 (0.72, 1.23) | -0.062 (-0.137, -0.008) |
| SO_2_ (0.5 ppb) | | 0.94 (0.79, 1.11) | 0.98 (0.84, 1.14) | 0.97 (0.74, 1.27) | | 0.91 (0.72, 1.15) | 0.004 (-0.037, 0.046) |
| Ovulation (0-5 days before sample) | | |  | |  |  |  |
| PM_2.5_ (5 μg/m^3^) | 1.12 (0.91, 1.39) | | 1.12 (0.96, 1.30) | | 1.14 (0.76, 1.71) | 1.17 (0.90, 1.52) | 0.024 (-0.034, 0.086) |
| NO_2_ (5 ppb) | 0.89 (0.68, 1.17) | | 1.10 (0.95, 1.26) | | 1.00 (0.61, 1.65) | 0.88 (0.62, 1.23) | -0.007 (-0.098, 0.067) |
| O_3_ (5 ppb) | 0.83 (0.70, 0.99) | | 0.88 (0.78, 0.99) | | 0.82 (0.64, 1.05) | 0.86 (0.67, 1.11) | -0.035 (-0.088, 0.007) |
| SO_2_ (0.5 ppb) | 0.92 (0.77, 1.10) | | 0.96 (0.82, 1.13) | | 0.96 (0.74, 1.26) | 0.88 (0.69, 1.12) | 0.002 (-0.030, 0.035) |
| Pre-implantation (1-5 days after sample) | | |  | |  |  |  |
| PM_2.5_ (5 μg/m^3^) | 1.08 (0.89, 1.30) | | 1.08 (0.93, 1.24) | | 1.19 (0.87, 1.62) | 1.04 (0.81, 1.33) | 0.040 (-0.015, 0.104) |
| NO_2_ (5 ppb) | 0.95 (0.73, 1.25) | | 1.06 (0.92, 1.22) | | 0.96 (0.58, 1.60) | 1.00 (0.70, 1.42) | -0.017 (-0.107, 0.055) |
| O_3_ (5 ppb) | 0.93 (0.78, 1.10) | | 0.94 (0.84, 1.05) | | 0.89 (0.71, 1.12) | 0.95 (0.74, 1.23) | -0.031 (-0.085, 0.012) |
| SO_2_ (0.5 ppb) | 0.96 (0.82, 1.12) | | 0.99 (0.85, 1.14) | | 0.98 (0.76, 1.26) | 0.94 (0.76, 1.16) | 0.002 (-0.031, 0.038) |
| Implantation (6-10 days after sample) | | |  | |  |  |  |
| PM_2.5_ (5 μg/m^3^) | 1.11 (0.90, 1.37) | | 1.08 (0.91, 1.27) | | 1.41 (0.99, 2.02) | 1.04 (0.79, 1.36) | 0.045 (-0.029, 0.128) |
| NO_2_ (5 ppb) | 0.89 (0.67, 1.17) | | 1.04 (0.90, 1.21) | | 0.86 (0.53, 1.38) | 0.99 (0.69, 1.42) | -0.029 (-0.111, 0.039) |
| O_3_ (5 ppb) | 0.87 (0.74, 1.03) | | 0.93 (0.83, 1.03) | | 0.79 (0.62, 1.00) | 0.94 (0.73, 1.20) | -0.035 (-0.091, 0.008) |
| SO_2_ (0.5 ppb) | 0.91 (0.77, 1.09) | | 0.93 (0.80, 1.09) | | 0.98 (0.77, 1.24) | 0.81 (0.61, 1.08) | 0.018 (-0.014, 0.055) |
| Post-implantation (11-14 days after sample) | | |  | |  |  |  |
| PM_2.5_ (5 μg/m^3^) | 1.03 (0.85, 1.25) | | 1.09 (0.94, 1.27) | | 1.15 (0.80, 1.65) | 1.04 (0.82, 1.31) | 0.039 (-0.021, 0.110) |
| NO_2_ (5 ppb) | 1.14 (0.88, 1.48) | | 1.10 (0.96, 1.26) | | 1.19 (0.78, 1.82) | 1.22 (0.86, 1.74) | -0.016 (-0.104, 0.056) |
| O_3_ (5 ppb) | 1.04 (0.89, 1.22) | | 0.97 (0.87, 1.07) | | 0.96 (0.78, 1.19) | 1.15 (0.90, 1.47) | -0.037 (-0.082, 0.000) |
| SO_2_ (0.5 ppb) | 0.97 (0.83, 1.13) | | 1.01 (0.88, 1.16) | | 0.99 (0.80, 1.23) | 0.91 (0.72, 1.15) | 0.013 (-0.021, 0.049) |

PM_2.5_: Particulate matter <2.5 microns; NO_2_: Nitrogen dioxide; O_3_: Ozone; SO_2_: Sulfur dioxide. All models adjust for male age, season, and income. Multipollutant models additionally adjust for co-pollutants PM_2.5_, NO_2_, O_3_, and SO_2_.

Table S3. Association of decile change in the air pollution mixture and hCG+ pregnancy, pregnancy loss, and live birth in intrauterine insemination (IUI) cycles (n=1,176).

|  | Mixture Model (quantile g-computation) | | |
| --- | --- | --- | --- |
|  | Overall | By season | |
|  |  | Warm season | Cold season |
|  | RR (95% CI) | RR (95% CI) | RR (95% CI) |
| **hCG+ pregnancy** |  |  |  |
| Spermatogenesis (0-74 days before sample) | 0.79 (0.62, 1.01) | 0.93 (0.78, 1.11) | 0.57 (0.38, 0.86) |
| Developmental windows |  |  |  |
| Mitosis (59-74 days before sample) | 0.83 (0.70, 0.99) | 0.99 (0.84, 1.17) | 0.70 (0.49, 0.98) |
| Meiosis (33-58 days before sample) | 0.84 (0.67, 1.06) | 1.01 (0.83, 1.22) | 0.62 (0.44, 0.87) |
| Spermiogenesis (11-32 days before sample) | 0.91 (0.72, 1.14) | 0.97 (0.82, 1.13) | 0.81 (0.57, 1.16) |
| Spermiation (0-10 days before sample) | 0.81 (0.68, 0.96) | 0.88 (0.75, 1.04) | 0.97 (0.68, 1.39) |
| Female partner windows |  |  |  |
| Ovulation (0-5 days before sample) | 0.90 (0.77, 1.06) | 0.92 (0.81, 1.04) | 0.92 (0.68, 1.23) |
| Pre-implantation (1-5 days after sample) | 0.99 (0.83, 1.17) | 0.99 (0.85, 1.14) | 1.14 (0.87, 1.49) |
| Implantation (6-10 days after sample) | 0.88 (0.73, 1.06) | 0.95 (0.81, 1.11) | 0.89 (0.68, 1.17) |
| Post-implantation (11-14 days after sample) | 1.04 (0.87, 1.24) | 1.00 (0.83, 1.20) | 1.05 (0.81, 1.37) |
| **Pregnancy loss (n=127)^a^** |  |  |  |
| Spermatogenesis (0-74 days before sample) | 0.97 (0.64, 1.48) |  |  |
| Developmental windows |  |  |  |
| Mitosis (59-74 days before sample) | 1.02 (0.73, 1.43) |  |  |
| Meiosis (33-58 days before sample) | 1.05 (0.69, 1.60) |  |  |
| Spermiogenesis (11-32 days before sample) | 1.02 (0.66, 1.58) |  |  |
| Spermiation (0-10 days before sample) | 0.99 (0.70, 1.41) |  |  |
| Female partner windows |  |  |  |
| Ovulation (0-5 days before sample) | 0.94 (0.66, 1.33) |  |  |
| Pre-implantation (1-5 days after sample) | 0.92 (0.67, 1.27) |  |  |
| Implantation (6-10 days after sample) | 1.09 (0.80, 1.48) |  |  |
| Post-implantation (11-14 days after sample) | 0.99 (0.77, 1.28) |  |  |
| **Live birth** |  |  |  |
| Spermatogenesis (0-74 days before sample) | 0.81 (0.6, 1.09) | 0.97 (0.78, 1.21) | 0.58 (0.35, 0.97) |
| Developmental windows |  |  |  |
| Mitosis (59-74 days before sample) | 0.91 (0.73, 1.13) | 1.06 (0.88, 1.28) | 0.78 (0.50, 1.20) |
| Meiosis (33-58 days before sample) | 0.84 (0.63, 1.11) | 1.07 (0.86, 1.32) | 0.58 (0.35, 0.94) |
| Spermiogenesis (11-32 days before sample) | 0.89 (0.68, 1.17) | 0.93 (0.74, 1.17) | 0.89 (0.57, 1.39) |
| Spermiation (0-10 days before sample) | 0.80 (0.61, 1.06) | 0.87 (0.71, 1.07) | 0.90 (0.57, 1.42) |
| Female partner windows |  |  |  |
| Ovulation (0-5 days before sample) | 0.88 (0.71, 1.08) | 0.92 (0.76, 1.10) | 0.83 (0.54, 1.28) |
| Pre-implantation (1-5 days after sample) | 1.01 (0.81, 1.27) | 0.98 (0.79, 1.20) | 1.07 (0.76, 1.52) |
| Implantation (6-10 days after sample) | 0.79 (0.60, 1.03) | 0.87 (0.69, 1.10) | 0.75 (0.47, 1.19) |
| Post-implantation (11-14 days after sample) | 1.01 (0.81, 1.25) | 0.97 (0.76, 1.24) | 1.09 (0.77, 1.55) |

All models adjust for male age, season, and income.

^a^Inverse probability weighting was used for multi-pollutant and single pregnancy loss models.

Table S4. Association of air pollution during spermatogenesis, ovulation, implantation, and live birth in intrauterine insemination (IUI) cycles: multipollutant model, single-pollutant model, and effect modification by season (multipollutant) (n=1,176).

|  | Live birth | | | | |
| --- | --- | --- | --- | --- | --- |
|  | Overall | | By season | | |
|  | Multipollutant | Single-pollutant | Multipollutant | | |
|  |  |  | Warm season | Cold season | RERI |
|  | RR (95% CI) | RR (95% CI) | RR (95% CI) | RR (95% CI) | Estimate (95% CI) |
| Spermatogenesis (0-74 days before sample) | |  |  |  |  |
| PM_2.5_ (5 μg/m^3^) | 1.22 (0.79, 1.89) | 1.26 (0.91, 1.75) | 1.50 (0.73, 3.08) | 1.14 (0.64, 2.05) | 0.059 (0.003, 0.129) |
| NO_2_ (5 ppb) | 0.82 (0.52, 1.29) | 1.15 (0.94, 1.41) | 1.28 (0.58, 2.85) | 0.68 (0.38, 1.24) | -0.014 (-0.106, 0.049) |
| O_3_ (5 ppb) | 0.76 (0.55, 1.06) | 0.84 (0.71, 1.00) | 0.91 (0.57, 1.43) | 0.66 (0.40, 1.08) | -0.024 (-0.090, 0.027) |
| SO_2_ (0.5 ppb) | 0.96 (0.77, 1.20) | 0.99 (0.81, 1.21) | 0.79 (0.53, 1.19) | 1.03 (0.79, 1.33) | -0.005 (-0.027, 0.014) |
| Mitosis (59-74 days before sample) | |  |  |  |  |
| PM_2.5_ (5 μg/m^3^) | 1.34 (1.00, 1.80) | 1.24 (1.02, 1.52) | 1.55 (1.05, 2.29) | 1.16 (0.77, 1.75) | 0.054 (0.001, 0.120) |
| NO_2_ (5 ppb) | 0.79 (0.54, 1.16) | 1.12 (0.96, 1.32) | 0.92 (0.47, 1.79) | 0.78 (0.47, 1.29) | -0.012 (-0.082, 0.042) |
| O_3_ (5 ppb) | 0.83 (0.66, 1.04) | 0.90 (0.80, 1.01) | 0.92 (0.66, 1.30) | 0.78 (0.57, 1.08) | -0.008 (-0.048, 0.023) |
| SO_2_ (0.5 ppb) | 1.00 (0.83, 1.21) | 1.03 (0.87, 1.22) | 0.88 (0.62, 1.26) | 1.05 (0.85, 1.29) | -0.005 (-0.023, 0.014) |
| Meiosis I+II (33-58 days before sample) | |  |  |  |  |
| PM_2.5_ (5 μg/m^3^) | 1.06 (0.74, 1.52) | 1.14 (0.88, 1.48) | 1.12 (0.58, 2.16) | 1.07 (0.70, 1.64) | 0.015 (-0.046, 0.077) |
| NO_2_ (5 ppb) | 0.94 (0.62, 1.43) | 1.16 (0.97, 1.39) | 1.57 (0.74, 3.30) | 0.75 (0.45, 1.24) | -0.007 (-0.079, 0.047) |
| O_3_ (5 ppb) | 0.82 (0.62, 1.09) | 0.85 (0.73, 0.99) | 1.04 (0.69, 1.57) | 0.67 (0.44, 1.01) | -0.017 (-0.069, 0.020) |
| SO_2_ (0.5 ppb) | 0.93 (0.74, 1.16) | 0.96 (0.78, 1.19) | 0.80 (0.53, 1.20) | 0.97 (0.74, 1.28) | 0.003 (-0.018, 0.024) |
| Spermiogenesis (11-32 days before sample) | |  |  |  |  |
| PM_2.5_ (5 μg/m^3^) | 1.03 (0.72, 1.48) | 1.10 (0.84, 1.43) | 1.20 (0.67, 2.15) | 1.03 (0.64, 1.68) | 0.033 (-0.022, 0.082) |
| NO_2_ (5 ppb) | 0.96 (0.65, 1.43) | 1.10 (0.91, 1.33) | 1.47 (0.72, 3.02) | 0.88 (0.53, 1.46) | 0.004 (-0.064, 0.057) |
| O_3_ (5 ppb) | 0.86 (0.65, 1.15) | 0.89 (0.75, 1.05) | 0.91 (0.61, 1.35) | 0.85 (0.55, 1.31) | -0.016 (-0.065, 0.025) |
| SO_2_ (0.5 ppb) | 0.96 (0.78, 1.18) | 0.99 (0.81, 1.20) | 0.77 (0.52, 1.16) | 1.03 (0.81, 1.31) | -0.007 (-0.028, 0.014) |
| Spermiation (0-10 days before sample) | |  |  |  |  |
| PM_2.5_ (5 μg/m^3^) | 1.02 (0.76, 1.37) | 1.02 (0.82, 1.28) | 1.03 (0.59, 1.77) | 1.09 (0.77, 1.55) | 0.021 (-0.013, 0.062) |
| NO_2_ (5 ppb) | 0.89 (0.63, 1.27) | 1.03 (0.86, 1.24) | 1.19 (0.61, 2.29) | 0.85 (0.55, 1.31) | -0.010 (-0.071, 0.040) |
| O_3_ (5 ppb) | 0.85 (0.68, 1.08) | 0.92 (0.79, 1.07) | 0.84 (0.60, 1.17) | 0.90 (0.64, 1.28) | -0.021 (-0.057, 0.01) |
| SO_2_ (0.5 ppb) | 0.96 (0.77, 1.19) | 0.97 (0.80, 1.18) | 0.82 (0.56, 1.20) | 1.03 (0.80, 1.32) | -0.008 (-0.03, 0.015) |
| Ovulation (0-5 days before sample) | |  |  |  |  |
| PM_2.5_ (5 μg/m^3^) | 1.13 (0.87, 1.47) | 1.10 (0.92, 1.33) | 1.15 (0.71, 1.87) | 1.20 (0.86, 1.67) | 0.021 (-0.015, 0.060) |
| NO_2_ (5 ppb) | 0.87 (0.62, 1.22) | 1.07 (0.90, 1.28) | 1.15 (0.64, 2.09) | 0.80 (0.53, 1.22) | -0.006 (-0.071, 0.047) |
| O_3_ (5 ppb) | 0.85 (0.68, 1.05) | 0.90 (0.78, 1.03) | 0.85 (0.62, 1.15) | 0.87 (0.63, 1.19) | -0.018 (-0.054, 0.010) |
| SO_2_ (0.5 ppb) | 0.94 (0.76, 1.17) | 0.98 (0.80, 1.19) | 0.84 (0.58, 1.21) | 1.00 (0.77, 1.30) | -0.007 (-0.027, 0.011) |
| Pre-implantation (1-5 days after sample) | |  |  |  |  |
| PM_2.5_ (5 μg/m^3^) | 1.16 (0.93, 1.44) | 1.12 (0.94, 1.32) | 1.11 (0.75, 1.66) | 1.21 (0.93, 1.59) | -0.003 (-0.046, 0.043) |
| NO_2_ (5 ppb) | 0.92 (0.66, 1.27) | 1.04 (0.87, 1.25) | 1.23 (0.66, 2.29) | 0.83 (0.55, 1.25) | 0.017 (-0.038, 0.065) |
| O_3_ (5 ppb) | 0.95 (0.77, 1.17) | 0.96 (0.84, 1.11) | 0.99 (0.74, 1.31) | 0.93 (0.68, 1.27) | -0.004 (-0.035, 0.022) |
| SO_2_ (0.5 ppb) | 0.95 (0.78, 1.17) | 0.98 (0.81, 1.18) | 0.85 (0.60, 1.22) | 1.00 (0.78, 1.29) | -0.007 (-0.028, 0.012) |
| Implantation (6-10 days after sample) | |  |  |  |  |
| PM_2.5_ (5 μg/m^3^) | 1.01 (0.75, 1.35) | 0.95 (0.75, 1.21) | 1.32 (0.82, 2.13) | 0.92 (0.62, 1.37) | 0.004 (-0.056, 0.065) |
| NO_2_ (5 ppb) | 0.81 (0.57, 1.15) | 0.97 (0.81, 1.18) | 0.85 (0.47, 1.54) | 0.83 (0.52, 1.31) | -0.017 (-0.084, 0.031) |
| O_3_ (5 ppb) | 0.81 (0.66, 0.99) | 0.92 (0.81, 1.05) | 0.75 (0.56, 1.00) | 0.84 (0.62, 1.15) | -0.016 (-0.055, 0.012) |
| SO_2_ (0.5 ppb) | 0.90 (0.71, 1.13) | 0.89 (0.72, 1.10) | 0.83 (0.58, 1.18) | 0.94 (0.69, 1.27) | -0.003 (-0.019, 0.011) |
| Post-implantation (11-14 days after sample) | |  |  |  |  |
| PM_2.5_ (5 μg/m^3^) | 0.79 (0.58, 1.08) | 0.90 (0.70, 1.15) | 1.11 (0.69, 1.79) | 0.70 (0.45, 1.09) | 0.026 (-0.014, 0.075) |
| NO_2_ (5 ppb) | 1.28 (0.91, 1.78) | 1.04 (0.88, 1.24) | 1.15 (0.69, 1.93) | 1.56 (0.99, 2.45) | -0.003 (-0.056, 0.042) |
| O_3_ (5 ppb) | 1.08 (0.89, 1.31) | 0.99 (0.88, 1.13) | 0.91 (0.70, 1.19) | 1.31 (0.96, 1.78) | -0.018 (-0.048, 0.005) |
| SO_2_ (0.5 ppb) | 0.96 (0.79, 1.17) | 0.99 (0.83, 1.18) | 0.90 (0.67, 1.22) | 0.98 (0.76, 1.27) | -0.003 (-0.024, 0.017) |

PM_2.5_: Particulate matter <2.5 microns; NO_2_: Nitrogen dioxide; O_3_: Ozone; SO_2_: Sulfur dioxide. All models adjust for male age, season, and income. Multipollutant models additionally adjust for co-pollutants PM_2.5_, NO_2_, O_3_, and SO_2_.

Table S5. Association of air pollution during spermatogenesis, ovulation, implantation, and pregnancy loss^a^ in intrauterine insemination (IUI) cycles: multipollutant and single-pollutant models (among n=127 with an observed pregnancy).

|  | Pregnancy loss | |
| --- | --- | --- |
|  | Overall | |
|  | Multipollutant | Single-pollutant |
|  | RR (95% CI) | RR (95% CI) |
| Spermatogenesis (0-74 days before sample) |  |  |
| PM_2.5_ (5 μg/m^3^) | 0.98 (0.53, 1.80) | 1.02 (0.71, 1.46) |
| NO_2_ (5 ppb) | 0.89 (0.49, 1.60) | 0.98 (0.77, 1.23) |
| O_3_ (5 ppb) | 0.90 (0.61, 1.35) | 0.99 (0.81, 1.20) |
| SO_2_ (0.5 ppb) | 1.03 (0.80, 1.32) | 0.99 (0.80, 1.23) |
| Mitosis (59-74 days before sample) |  |  |
| PM_2.5_ (5 μg/m^3^) | 0.87 (0.55, 1.40) | 0.84 (0.65, 1.10) |
| NO_2_ (5 ppb) | 0.90 (0.52, 1.56) | 0.90 (0.73, 1.11) |
| O_3_ (5 ppb) | 0.96 (0.74, 1.26) | 1.02 (0.90, 1.17) |
| SO_2_ (0.5 ppb) | 1.06 (0.84, 1.33) | 0.96 (0.78, 1.18) |
| Meiosis I+II (33-58 days before sample) |  |  |
| PM_2.5_ (5 μg/m^3^) | 1.20 (0.77, 1.85) | 1.05 (0.81, 1.36) |
| NO_2_ (5 ppb) | 0.80 (0.49, 1.32) | 0.94 (0.77, 1.15) |
| O_3_ (5 ppb) | 0.95 (0.69, 1.31) | 1.03 (0.87, 1.22) |
| SO_2_ (0.5 ppb) | 1.06 (0.82, 1.37) | 0.99 (0.78, 1.25) |
| Spermiogenesis (11-32 days before sample) |  |  |
| PM_2.5_ (5 μg/m^3^) | 0.92 (0.58, 1.47) | 0.98 (0.72, 1.33) |
| NO_2_ (5 ppb) | 0.98 (0.59, 1.62) | 1.01 (0.82, 1.25) |
| O_3_ (5 ppb) | 0.91 (0.63, 1.30) | 0.97 (0.80, 1.17) |
| SO_2_ (0.5 ppb) | 1.00 (0.81, 1.25) | 0.99 (0.82, 1.20) |
| Spermiation (0-10 days before sample) |  |  |
| PM_2.5_ (5 μg/m^3^) | 0.98 (0.70, 1.38) | 1.15 (0.98, 1.35) |
| NO_2_ (5 ppb) | 1.04 (0.69, 1.58) | 1.17 (0.98, 1.38) |
| O_3_ (5 ppb) | 0.87 (0.67, 1.11) | 0.88 (0.74, 1.04) |
| SO_2_ (0.5 ppb) | 0.96 (0.75, 1.24) | 1.05 (0.85, 1.28) |
| Ovulation (0-5 days before sample) |  |  |
| PM_2.5_ (5 μg/m^3^) | 0.87 (0.60, 1.24) | 1.04 (0.86, 1.26) |
| NO_2_ (5 ppb) | 1.11 (0.73, 1.70) | 1.08 (0.90, 1.30) |
| O_3_ (5 ppb) | 0.92 (0.72, 1.18) | 0.93 (0.79, 1.10) |
| SO_2_ (0.5 ppb) | 0.97 (0.74, 1.26) | 1.00 (0.81, 1.24) |
| Pre-implantation (1-5 days after sample) |  |  |
| PM_2.5_ (5 μg/m^3^) | 0.90 (0.67, 1.21) | 0.92 (0.75, 1.12) |
| NO_2_ (5 ppb) | 1.03 (0.75, 1.40) | 1.04 (0.87, 1.26) |
| O_3_ (5 ppb) | 0.90 (0.72, 1.12) | 0.94 (0.81, 1.09) |
| SO_2_ (0.5 ppb) | 1.01 (0.80, 1.27) | 1.02 (0.84, 1.24) |
| Implantation (6-10 days after sample) |  |  |
| PM_2.5_ (5 μg/m^3^) | 1.12 (0.90, 1.40) | 1.13 (0.97, 1.33) |
| NO_2_ (5 ppb) | 1.09 (0.77, 1.54) | 1.13 (0.96, 1.33) |
| O_3_ (5 ppb) | 1.01 (0.80, 1.27) | 0.98 (0.85, 1.13) |
| SO_2_ (0.5 ppb) | 1.03 (0.82, 1.28) | 1.11 (0.90, 1.36) |
| Post-implantation (11-14 days after sample) |  |  |
| PM_2.5_ (5 μg/m^3^) | 1.21 (1.08, 1.36) | 1.19 (1.07, 1.32) |
| NO_2_ (5 ppb) | 0.95 (0.69, 1.30) | 1.10 (0.94, 1.29) |
| O_3_ (5 ppb) | 0.91 (0.74, 1.13) | 0.94 (0.83, 1.07) |
| SO_2_ (0.5 ppb) | 1.01 (0.85, 1.20) | 1.04 (0.88, 1.23) |

PM_2.5_: Particulate matter <2.5 microns; NO_2_: Nitrogen dioxide; O_3_: Ozone; SO_2_: Sulfur dioxide. All models adjust for male age, season, and income. Multipollutant models additionally adjust for co-pollutants PM_2.5_, NO_2_, O_3_, and SO_2_.

^a^Inverse probability weighting was used for multi-pollutant and single pregnancy loss models.

Table S6. Association of air pollution with pregnancy, pregnancy loss^a^, and live birth in intrauterine insemination (IUI) cycles additionally adjusted for female partner age: multipollutant and single-pollutant models (n=1,176).

|  | Models additionally adjusted for female partner age | | | | | |
| --- | --- | --- | --- | --- | --- | --- |
|  | Pregnancy | | Pregnancy loss | | Live birth | |
|  | Multipollutant | Single-pollutant | Multipollutant | Single-pollutant | Multipollutant | Single-pollutant |
|  | RR (95% CI) | RR (95% CI) | RR (95% CI) | RR (95% CI) | RR (95% CI) | RR (95% CI) |
| Spermatogenesis (0-74 days before sample) | | |  |  |  |  |
| PM_2.5_ (5 μg/m^3^) | 1.31 (0.93, 1.86) | 1.28 (0.99, 1.66) | 0.98 (0.53, 1.81) | 1.02 (0.71, 1.46) | 1.23 (0.79, 1.90) | 1.27 (0.92, 1.76) |
| NO_2_ (5 ppb) | 0.73 (0.51, 1.05) | 1.13 (0.96, 1.33) | 0.90 (0.49, 1.65) | 0.98 (0.77, 1.23) | 0.82 (0.52, 1.29) | 1.16 (0.94, 1.42) |
| O_3_ (5 ppb) | 0.71 (0.55, 0.93) | 0.84 (0.73, 0.96) | 0.91 (0.61, 1.36) | 0.99 (0.81, 1.20) | 0.76 (0.55, 1.06) | 0.84 (0.71, 1.00) |
| SO_2_ (0.5 ppb) | 0.96 (0.80, 1.14) | 0.97 (0.82, 1.15) | 1.02 (0.80, 1.31) | 0.99 (0.79, 1.23) | 0.96 (0.77, 1.20) | 0.99 (0.81, 1.21) |
| Mitosis (59-74 days before sample) | |  |  |  |  |  |
| PM_2.5_ (5 μg/m^3^) | 1.32 (1.03, 1.68) | 1.16 (0.98, 1.38) | 0.86 (0.53, 1.39) | 0.84 (0.65, 1.10) | 1.34 (1.00, 1.80) | 1.25 (1.02, 1.53) |
| NO_2_ (5 ppb) | 0.70 (0.51, 0.96) | 1.07 (0.94, 1.22) | 0.92 (0.52, 1.62) | 0.90 (0.73, 1.11) | 0.79 (0.54, 1.16) | 1.13 (0.96, 1.32) |
| O_3_ (5 ppb) | 0.78 (0.65, 0.93) | 0.91 (0.82, 1.00) | 0.97 (0.73, 1.27) | 1.02 (0.90, 1.17) | 0.83 (0.67, 1.05) | 0.90 (0.80, 1.01) |
| SO_2_ (0.5 ppb) | 1.00 (0.85, 1.17) | 1.00 (0.86, 1.15) | 1.05 (0.83, 1.32) | 0.96 (0.78, 1.18) | 1.00 (0.83, 1.21) | 1.03 (0.87, 1.22) |
| Meiosis I+II (33-58 days before sample) | | |  |  |  |  |
| PM_2.5_ (5 μg/m^3^) | 1.20 (0.92, 1.57) | 1.18 (0.96, 1.45) | 1.20 (0.77, 1.86) | 1.05 (0.81, 1.36) | 1.06 (0.75, 1.52) | 1.15 (0.88, 1.49) |
| NO_2_ (5 ppb) | 0.80 (0.58, 1.11) | 1.12 (0.97, 1.29) | 0.81 (0.49, 1.34) | 0.94 (0.76, 1.15) | 0.95 (0.63, 1.43) | 1.16 (0.97, 1.39) |
| O_3_ (5 ppb) | 0.77 (0.61, 0.96) | 0.86 (0.76, 0.97) | 0.96 (0.69, 1.32) | 1.03 (0.87, 1.22) | 0.82 (0.62, 1.09) | 0.85 (0.73, 0.99) |
| SO_2_ (0.5 ppb) | 0.93 (0.78, 1.12) | 0.95 (0.80, 1.13) | 1.05 (0.82, 1.36) | 0.99 (0.78, 1.25) | 0.93 (0.74, 1.17) | 0.97 (0.78, 1.19) |
| Spermiogenesis (11-32 days before sample) | | |  |  |  |  |
| PM_2.5_ (5 μg/m^3^) | 1.04 (0.78, 1.39) | 1.10 (0.89, 1.37) | 0.93 (0.58, 1.48) | 0.98 (0.72, 1.32) | 1.03 (0.72, 1.48) | 1.10 (0.84, 1.44) |
| NO_2_ (5 ppb) | 0.95 (0.69, 1.30) | 1.10 (0.94, 1.28) | 0.99 (0.59, 1.66) | 1.01 (0.81, 1.25) | 0.96 (0.65, 1.42) | 1.10 (0.91, 1.33) |
| O_3_ (5 ppb) | 0.85 (0.67, 1.07) | 0.88 (0.77, 1.01) | 0.90 (0.63, 1.30) | 0.97 (0.80, 1.17) | 0.86 (0.65, 1.15) | 0.89 (0.75, 1.05) |
| SO_2_ (0.5 ppb) | 0.95 (0.80, 1.12) | 0.98 (0.84, 1.14) | 1.00 (0.80, 1.24) | 0.99 (0.81, 1.20) | 0.96 (0.78, 1.18) | 0.99 (0.82, 1.20) |
| Spermiation (0-10 days before sample) | |  |  |  |  |  |
| PM_2.5_ (5 μg/m^3^) | 1.10 (0.88, 1.37) | 1.12 (0.95, 1.32) | 0.98 (0.68, 1.40) | 1.15 (0.98, 1.36) | 1.02 (0.76, 1.37) | 1.02 (0.82, 1.28) |
| NO_2_ (5 ppb) | 0.91 (0.69, 1.19) | 1.10 (0.96, 1.27) | 1.08 (0.70, 1.64) | 1.17 (0.98, 1.40) | 0.89 (0.63, 1.27) | 1.03 (0.86, 1.24) |
| O_3_ (5 ppb) | 0.83 (0.69, 1.01) | 0.87 (0.77, 0.99) | 0.86 (0.67, 1.11) | 0.87 (0.73, 1.04) | 0.85 (0.67, 1.08) | 0.92 (0.79, 1.07) |
| SO_2_ (0.5 ppb) | 0.94 (0.79, 1.11) | 0.98 (0.84, 1.14) | 0.94 (0.74, 1.21) | 1.05 (0.85, 1.28) | 0.96 (0.77, 1.19) | 0.97 (0.80, 1.18) |
| Ovulation (0-5 days before sample) | |  |  |  |  |  |
| PM_2.5_ (5 μg/m^3^) | 1.13 (0.91, 1.40) | 1.12 (0.97, 1.30) | 0.86 (0.59, 1.25) | 1.04 (0.86, 1.26) | 1.13 (0.87, 1.48) | 1.11 (0.92, 1.34) |
| NO_2_ (5 ppb) | 0.89 (0.68, 1.16) | 1.10 (0.96, 1.26) | 1.15 (0.74, 1.79) | 1.08 (0.90, 1.31) | 0.87 (0.62, 1.22) | 1.08 (0.90, 1.28) |
| O_3_ (5 ppb) | 0.83 (0.70, 0.99) | 0.88 (0.78, 0.98) | 0.92 (0.72, 1.18) | 0.93 (0.78, 1.10) | 0.84 (0.68, 1.05) | 0.89 (0.78, 1.03) |
| SO_2_ (0.5 ppb) | 0.92 (0.77, 1.10) | 0.97 (0.82, 1.13) | 0.94 (0.72, 1.23) | 1.00 (0.81, 1.24) | 0.94 (0.76, 1.17) | 0.98 (0.81, 1.19) |
| Pre-implantation (1-5 days after sample) | | |  |  |  |  |
| PM_2.5_ (5 μg/m^3^) | 1.08 (0.89, 1.30) | 1.08 (0.93, 1.24) | 0.89 (0.67, 1.20) | 0.92 (0.75, 1.12) | 1.16 (0.93, 1.44) | 1.12 (0.94, 1.32) |
| NO_2_ (5 ppb) | 0.95 (0.73, 1.25) | 1.06 (0.92, 1.23) | 1.05 (0.77, 1.44) | 1.04 (0.86, 1.26) | 0.91 (0.66, 1.27) | 1.04 (0.87, 1.25) |
| O_3_ (5 ppb) | 0.92 (0.78, 1.09) | 0.94 (0.84, 1.05) | 0.89 (0.71, 1.11) | 0.94 (0.81, 1.09) | 0.95 (0.77, 1.16) | 0.96 (0.84, 1.10) |
| SO_2_ (0.5 ppb) | 0.96 (0.82, 1.13) | 0.99 (0.86, 1.14) | 0.99 (0.78, 1.25) | 1.02 (0.84, 1.24) | 0.96 (0.78, 1.17) | 0.98 (0.81, 1.18) |
| Implantation (6-10 days after sample) | |  |  |  |  |  |
| PM_2.5_ (5 μg/m^3^) | 1.11 (0.90, 1.37) | 1.08 (0.92, 1.28) | 1.12 (0.90, 1.40) | 1.13 (0.97, 1.32) | 1.01 (0.76, 1.36) | 0.95 (0.75, 1.21) |
| NO_2_ (5 ppb) | 0.89 (0.68, 1.17) | 1.05 (0.91, 1.21) | 1.11 (0.78, 1.57) | 1.13 (0.95, 1.34) | 0.81 (0.57, 1.15) | 0.98 (0.81, 1.18) |
| O_3_ (5 ppb) | 0.87 (0.74, 1.03) | 0.92 (0.83, 1.02) | 1.00 (0.80, 1.26) | 0.98 (0.85, 1.13) | 0.81 (0.66, 0.99) | 0.92 (0.81, 1.05) |
| SO_2_ (0.5 ppb) | 0.91 (0.77, 1.09) | 0.94 (0.80, 1.10) | 1.01 (0.81, 1.27) | 1.11 (0.90, 1.36) | 0.90 (0.72, 1.13) | 0.89 (0.72, 1.10) |
| Post-implantation (11-14 days after sample) | | |  |  |  |  |
| PM_2.5_ (5 μg/m^3^) | 1.03 (0.85, 1.25) | 1.10 (0.94, 1.27) | 1.20 (1.07, 1.35) | 1.19 (1.07, 1.32) | 0.79 (0.58, 1.08) | 0.90 (0.70, 1.15) |
| NO_2_ (5 ppb) | 1.14 (0.88, 1.48) | 1.10 (0.96, 1.26) | 0.96 (0.69, 1.33) | 1.10 (0.94, 1.29) | 1.27 (0.91, 1.78) | 1.05 (0.88, 1.25) |
| O_3_ (5 ppb) | 1.04 (0.88, 1.21) | 0.96 (0.87, 1.07) | 0.90 (0.72, 1.13) | 0.94 (0.82, 1.07) | 1.07 (0.88, 1.31) | 0.99 (0.87, 1.13) |
| SO_2_ (0.5 ppb) | 0.97 (0.83, 1.13) | 1.01 (0.88, 1.16) | 0.99 (0.83, 1.18) | 1.04 (0.88, 1.23) | 0.96 (0.79, 1.17) | 0.99 (0.83, 1.18) |

PM_2.5_: Particulate matter <2.5 microns; NO_2_: Nitrogen dioxide; O_3_: Ozone; SO_2_: Sulfur dioxide. All models adjust for male age, season, income, and female partner age. Multipollutant models additionally adjust for co-pollutants PM_2.5_, NO_2_, O_3_, and SO_2_.

^a^Inverse probability weighting was used for multi-pollutant and single pregnancy loss models.

Table S7. Association of air pollution with pregnancy, pregnancy loss^a^, and live birth in intrauterine insemination (IUI) cycles additionally adjusted for self-reported prior male-factor infertility diagnosis: multipollutant and single-pollutant models (n=1,176).

|  | Models additionally adjusted for prior male-factor infertility diagnosis | | | | | |
| --- | --- | --- | --- | --- | --- | --- |
|  | Pregnancy  (n,=1,176) | | Pregnancy loss  (n=127) | | Live birth  (n=1,176) | |
|  | Multipollutant | Single-pollutant | Multipollutant | Single-pollutant | Multipollutant | Single-pollutant |
|  | RR (95% CI) | RR (95% CI) | RR (95% CI) | RR (95% CI) | RR (95% CI) | RR (95% CI) |
| Spermatogenesis (0-74 days before sample) | | |  |  |  |  |
| PM_2.5_ (5 μg/m^3^) | 1.30 (0.92, 1.84) | 1.29 (0.99, 1.67) | 1.03 (0.56, 1.90) | 1.02 (0.71, 1.46) | 1.22 (0.78, 1.89) | 1.28 (0.93, 1.78) |
| NO_2_ (5 ppb) | 0.75 (0.52, 1.08) | 1.14 (0.97, 1.35) | 0.85 (0.46, 1.56) | 0.97 (0.76, 1.24) | 0.84 (0.53, 1.34) | 1.17 (0.95, 1.44) |
| O_3_ (5 ppb) | 0.72 (0.55, 0.94) | 0.83 (0.73, 0.96) | 0.91 (0.61, 1.36) | 0.99 (0.81, 1.21) | 0.77 (0.55, 1.07) | 0.83 (0.70, 0.99) |
| SO_2_ (0.5 ppb) | 0.95 (0.80, 1.14) | 0.97 (0.83, 1.15) | 1.03 (0.80, 1.32) | 0.99 (0.79, 1.23) | 0.96 (0.77, 1.20) | 0.99 (0.81, 1.22) |
| Mitosis (59-74 days before sample) | |  |  |  |  |  |
| PM_2.5_ (5 μg/m^3^) | 1.31 (1.03, 1.67) | 1.17 (0.98, 1.39) | 0.90 (0.57, 1.44) | 0.84 (0.64, 1.10) | 1.33 (0.99, 1.79) | 1.25 (1.02, 1.54) |
| NO_2_ (5 ppb) | 0.71 (0.52, 0.98) | 1.08 (0.94, 1.23) | 0.85 (0.49, 1.47) | 0.89 (0.72, 1.11) | 0.81 (0.55, 1.18) | 1.13 (0.97, 1.33) |
| O_3_ (5 ppb) | 0.78 (0.65, 0.93) | 0.90 (0.82, 0.99) | 0.95 (0.73, 1.25) | 1.03 (0.89, 1.17) | 0.84 (0.67, 1.05) | 0.90 (0.80, 1.01) |
| SO_2_ (0.5 ppb) | 1.00 (0.85, 1.17) | 1.00 (0.86, 1.15) | 1.06 (0.84, 1.34) | 0.96 (0.78, 1.18) | 1.00 (0.83, 1.21) | 1.03 (0.87, 1.22) |
| Meiosis I+II (33-58 days before sample) | | |  |  |  |  |
| PM_2.5_ (5 μg/m^3^) | 1.20 (0.91, 1.57) | 1.18 (0.96, 1.46) | 1.26 (0.80, 1.99) | 1.05 (0.81, 1.37) | 1.05 (0.74, 1.51) | 1.15 (0.89, 1.50) |
| NO_2_ (5 ppb) | 0.82 (0.59, 1.13) | 1.13 (0.97, 1.31) | 0.77 (0.46, 1.29) | 0.93 (0.76, 1.15) | 0.97 (0.64, 1.47) | 1.18 (0.98, 1.41) |
| O_3_ (5 ppb) | 0.77 (0.62, 0.96) | 0.85 (0.75, 0.97) | 0.96 (0.69, 1.33) | 1.03 (0.87, 1.23) | 0.83 (0.62, 1.10) | 0.84 (0.72, 0.98) |
| SO_2_ (0.5 ppb) | 0.93 (0.78, 1.12) | 0.96 (0.81, 1.13) | 1.06 (0.82, 1.38) | 0.99 (0.78, 1.25) | 0.92 (0.73, 1.16) | 0.97 (0.79, 1.19) |
| Spermiogenesis (11-32 days before sample) | | |  |  |  |  |
| PM_2.5_ (5 μg/m^3^) | 1.03 (0.77, 1.38) | 1.11 (0.89, 1.37) | 0.94 (0.59, 1.51) | 0.98 (0.73, 1.33) | 1.02 (0.71, 1.47) | 1.11 (0.85, 1.45) |
| NO_2_ (5 ppb) | 0.97 (0.71, 1.34) | 1.11 (0.95, 1.30) | 0.95 (0.57, 1.61) | 1.01 (0.82, 1.25) | 0.99 (0.67, 1.47) | 1.12 (0.92, 1.35) |
| O_3_ (5 ppb) | 0.86 (0.68, 1.08) | 0.88 (0.76, 1.01) | 0.91 (0.63, 1.30) | 0.97 (0.80, 1.17) | 0.87 (0.65, 1.16) | 0.88 (0.74, 1.04) |
| SO_2_ (0.5 ppb) | 0.94 (0.80, 1.12) | 0.98 (0.84, 1.15) | 1.01 (0.81, 1.25) | 0.99 (0.82, 1.20) | 0.96 (0.78, 1.18) | 1.00 (0.82, 1.21) |
| Spermiation (0-10 days before sample) | |  |  |  |  |  |
| PM_2.5_ (5 μg/m^3^) | 1.09 (0.87, 1.37) | 1.12 (0.95, 1.32) | 0.99 (0.70, 1.40) | 1.15 (0.98, 1.35) | 1.02 (0.76, 1.37) | 1.03 (0.82, 1.29) |
| NO_2_ (5 ppb) | 0.92 (0.70, 1.22) | 1.11 (0.97, 1.29) | 1.03 (0.67, 1.57) | 1.17 (0.98, 1.39) | 0.91 (0.64, 1.30) | 1.05 (0.87, 1.26) |
| O_3_ (5 ppb) | 0.84 (0.69, 1.01) | 0.87 (0.77, 0.98) | 0.86 (0.67, 1.11) | 0.88 (0.74, 1.04) | 0.86 (0.68, 1.09) | 0.91 (0.78, 1.06) |
| SO_2_ (0.5 ppb) | 0.94 (0.79, 1.11) | 0.98 (0.84, 1.15) | 0.96 (0.74, 1.24) | 1.04 (0.85, 1.29) | 0.96 (0.77, 1.19) | 0.98 (0.8, 1.19) |
| Ovulation (0-5 days before sample) | |  |  |  |  |  |
| PM_2.5_ (5 μg/m^3^) | 1.12 (0.90, 1.39) | 1.13 (0.97, 1.31) | 0.87 (0.61, 1.25) | 1.04 (0.86, 1.27) | 1.13 (0.86, 1.47) | 1.12 (0.92, 1.35) |
| NO_2_ (5 ppb) | 0.91 (0.69, 1.19) | 1.11 (0.97, 1.28) | 1.10 (0.71, 1.70) | 1.08 (0.90, 1.31) | 0.89 (0.64, 1.25) | 1.09 (0.91, 1.30) |
| O_3_ (5 ppb) | 0.84 (0.70, 1.00) | 0.87 (0.78, 0.98) | 0.92 (0.71, 1.18) | 0.93 (0.79, 1.10) | 0.85 (0.68, 1.06) | 0.89 (0.77, 1.03) |
| SO_2_ (0.5 ppb) | 0.92 (0.77, 1.10) | 0.97 (0.83, 1.14) | 0.96 (0.73, 1.26) | 1.00 (0.80, 1.25) | 0.95 (0.76, 1.17) | 0.98 (0.81, 1.20) |
| Pre-implantation (1-5 days after sample) | | |  |  |  |  |
| PM_2.5_ (5 μg/m^3^) | 1.07 (0.88, 1.30) | 1.08 (0.94, 1.25) | 0.90 (0.67, 1.21) | 0.91 (0.75, 1.12) | 1.15 (0.93, 1.44) | 1.12 (0.95, 1.33) |
| NO_2_ (5 ppb) | 0.97 (0.74, 1.28) | 1.07 (0.93, 1.24) | 1.01 (0.73, 1.38) | 1.04 (0.86, 1.27) | 0.93 (0.67, 1.30) | 1.06 (0.88, 1.27) |
| O_3_ (5 ppb) | 0.93 (0.79, 1.10) | 0.94 (0.84, 1.04) | 0.89 (0.71, 1.11) | 0.94 (0.81, 1.09) | 0.95 (0.77, 1.17) | 0.96 (0.83, 1.10) |
| SO_2_ (0.5 ppb) | 0.96 (0.82, 1.13) | 0.99 (0.86, 1.15) | 1.01 (0.80, 1.27) | 1.01 (0.83, 1.24) | 0.96 (0.78, 1.18) | 0.98 (0.82, 1.18) |
| Implantation (6-10 days after sample) | |  |  |  |  |  |
| PM_2.5_ (5 μg/m^3^) | 1.10 (0.89, 1.35) | 1.08 (0.92, 1.27) | 1.13 (0.90, 1.41) | 1.13 (0.97, 1.33) | 1.00 (0.75, 1.34) | 0.95 (0.75, 1.21) |
| NO_2_ (5 ppb) | 0.91 (0.69, 1.21) | 1.06 (0.91, 1.23) | 1.08 (0.76, 1.55) | 1.13 (0.95, 1.34) | 0.82 (0.58, 1.18) | 0.99 (0.82, 1.20) |
| O_3_ (5 ppb) | 0.88 (0.74, 1.04) | 0.92 (0.83, 1.02) | 1.01 (0.80, 1.27) | 0.98 (0.85, 1.13) | 0.81 (0.66, 1.00) | 0.92 (0.80, 1.04) |
| SO_2_ (0.5 ppb) | 0.91 (0.77, 1.09) | 0.94 (0.80, 1.10) | 1.03 (0.83, 1.28) | 1.11 (0.90, 1.36) | 0.90 (0.72, 1.14) | 0.90 (0.73, 1.11) |
| Post-implantation (11-14 days after sample) | | |  |  |  |  |
| PM_2.5_ (5 μg/m^3^) | 1.02 (0.84, 1.24) | 1.10 (0.95, 1.27) | 1.21 (1.07, 1.36) | 1.19 (1.07, 1.32) | 0.78 (0.57, 1.08) | 0.90 (0.71, 1.16) |
| NO_2_ (5 ppb) | 1.17 (0.89, 1.52) | 1.11 (0.97, 1.28) | 0.94 (0.68, 1.30) | 1.10 (0.94, 1.29) | 1.31 (0.93, 1.83) | 1.06 (0.89, 1.26) |
| O_3_ (5 ppb) | 1.04 (0.89, 1.22) | 0.96 (0.87, 1.07) | 0.91 (0.73, 1.13) | 0.94 (0.83, 1.07) | 1.08 (0.89, 1.32) | 0.99 (0.87, 1.12) |
| SO_2_ (0.5 ppb) | 0.97 (0.83, 1.13) | 1.01 (0.88, 1.17) | 1.01 (0.84, 1.20) | 1.05 (0.88, 1.24) | 0.96 (0.79, 1.17) | 1.00 (0.84, 1.19) |

PM_2.5_: Particulate matter <2.5 microns; NO_2_: Nitrogen dioxide; O_3_: Ozone; SO_2_: Sulfur dioxide. All models adjust for male age, season, income, and prior diagnosis of male-factor infertility (yes versus no/missing). Multipollutant models additionally adjust for co-pollutants PM_2.5_, NO_2_, O_3_, and SO_2_.

^a^Inverse probability weighting was used for multi-pollutant and single pregnancy loss models.

Table S8. Association of air pollution with pregnancy, pregnancy loss^a^, and live birth in intrauterine insemination (IUI) cycles additionally adjusted for a baseline semen quality parameter below WHO 5% percentile: multipollutant and single-pollutant models.

|  | Models additionally adjusted for a baseline semen quality parameter <WHO 5^th^ percentile | | | | | |
| --- | --- | --- | --- | --- | --- | --- |
|  | Pregnancy  (n=1,158) | | Pregnancy loss  (n=127) | | Live birth  (n=1,158) | |
|  | Multipollutant | Single-pollutant | Multipollutant | Single-pollutant | Multipollutant | Single-pollutant |
|  | RR (95% CI) | RR (95% CI) | RR (95% CI) | RR (95% CI) | RR (95% CI) | RR (95% CI) |
| Spermatogenesis (0-74 days before sample) | | |  |  |  |  |
| PM_2.5_ (5 μg/m^3^) | 1.28 (0.90, 1.81) | 1.26 (0.97, 1.64) | 1.01 (0.55, 1.83) | 0.99 (0.69, 1.41) | 1.18 (0.76, 1.84) | 1.26 (0.91, 1.75) |
| NO_2_ (5 ppb) | 0.75 (0.52, 1.08) | 1.13 (0.96, 1.34) | 0.82 (0.46, 1.44) | 0.94 (0.75, 1.18) | 0.86 (0.54, 1.37) | 1.17 (0.95, 1.44) |
| O_3_ (5 ppb) | 0.72 (0.55, 0.94) | 0.84 (0.73, 0.96) | 0.90 (0.61, 1.32) | 1.01 (0.83, 1.22) | 0.77 (0.55, 1.08) | 0.83 (0.70, 0.99) |
| SO_2_ (0.5 ppb) | 0.95 (0.79, 1.14) | 0.97 (0.82, 1.14) | 1.06 (0.83, 1.36) | 0.99 (0.79, 1.24) | 0.95 (0.76, 1.19) | 0.98 (0.80, 1.21) |
| Mitosis (59-74 days before sample) | |  |  |  |  |  |
| PM_2.5_ (5 μg/m^3^) | 1.32 (1.03, 1.68) | 1.17 (0.98, 1.40) | 0.89 (0.55, 1.42) | 0.84 (0.64, 1.09) | 1.33 (0.99, 1.79) | 1.26 (1.03, 1.54) |
| NO_2_ (5 ppb) | 0.71 (0.51, 0.97) | 1.09 (0.95, 1.24) | 0.84 (0.49, 1.43) | 0.89 (0.72, 1.10) | 0.81 (0.55, 1.19) | 1.15 (0.98, 1.34) |
| O_3_ (5 ppb) | 0.77 (0.64, 0.93) | 0.90 (0.81, 0.99) | 0.94 (0.73, 1.22) | 1.03 (0.90, 1.17) | 0.83 (0.66, 1.04) | 0.89 (0.79, 1.00) |
| SO_2_ (0.5 ppb) | 0.99 (0.85, 1.16) | 0.99 (0.86, 1.15) | 1.09 (0.86, 1.39) | 0.97 (0.78, 1.20) | 1.00 (0.83, 1.20) | 1.03 (0.87, 1.22) |
| Meiosis I+II (33-58 days before sample) | | |  |  |  |  |
| PM_2.5_ (5 μg/m^3^) | 1.19 (0.91, 1.57) | 1.18 (0.96, 1.45) | 1.21 (0.81, 1.83) | 1.03 (0.80, 1.33) | 1.04 (0.73, 1.50) | 1.16 (0.89, 1.50) |
| NO_2_ (5 ppb) | 0.82 (0.60, 1.14) | 1.13 (0.97, 1.31) | 0.76 (0.47, 1.22) | 0.91 (0.74, 1.11) | 0.99 (0.65, 1.51) | 1.18 (0.99, 1.41) |
| O_3_ (5 ppb) | 0.77 (0.62, 0.97) | 0.85 (0.75, 0.97) | 0.96 (0.71, 1.30) | 1.05 (0.89, 1.24) | 0.83 (0.63, 1.11) | 0.84 (0.72, 0.98) |
| SO_2_ (0.5 ppb) | 0.93 (0.77, 1.12) | 0.95 (0.80, 1.13) | 1.10 (0.85, 1.42) | 0.99 (0.78, 1.25) | 0.92 (0.73, 1.16) | 0.96 (0.78, 1.19) |
| Spermiogenesis (11-32 days before sample) | | |  |  |  |  |
| PM_2.5_ (5 μg/m^3^) | 1.01 (0.76, 1.36) | 1.08 (0.87, 1.34) | 0.94 (0.60, 1.47) | 0.96 (0.71, 1.28) | 1.00 (0.69, 1.44) | 1.08 (0.82, 1.42) |
| NO_2_ (5 ppb) | 0.97 (0.70, 1.33) | 1.09 (0.94, 1.27) | 0.90 (0.55, 1.48) | 0.97 (0.79, 1.2) | 1.00 (0.67, 1.49) | 1.10 (0.91, 1.33) |
| O_3_ (5 ppb) | 0.86 (0.68, 1.09) | 0.89 (0.77, 1.02) | 0.89 (0.63, 1.26) | 0.99 (0.82, 1.19) | 0.88 (0.66, 1.18) | 0.89 (0.75, 1.06) |
| SO_2_ (0.5 ppb) | 0.94 (0.79, 1.12) | 0.97 (0.83, 1.14) | 1.02 (0.82, 1.28) | 0.98 (0.8, 1.2) | 0.95 (0.77, 1.17) | 0.98 (0.81, 1.19) |
| Spermiation (0-10 days before sample) | |  |  |  |  |  |
| PM_2.5_ (5 μg/m^3^) | 1.07 (0.85, 1.34) | 1.09 (0.93, 1.29) | 0.99 (0.72, 1.37) | 1.13 (0.97, 1.32) | 0.98 (0.73, 1.33) | 1.00 (0.79, 1.26) |
| NO_2_ (5 ppb) | 0.93 (0.70, 1.23) | 1.09 (0.95, 1.26) | 1.00 (0.66, 1.50) | 1.13 (0.95, 1.34) | 0.93 (0.65, 1.33) | 1.03 (0.86, 1.25) |
| O_3_ (5 ppb) | 0.85 (0.70, 1.02) | 0.88 (0.78, 1.00) | 0.87 (0.68, 1.11) | 0.90 (0.76, 1.06) | 0.87 (0.68, 1.10) | 0.92 (0.79, 1.07) |
| SO_2_ (0.5 ppb) | 0.93 (0.79, 1.11) | 0.97 (0.83, 1.14) | 0.99 (0.77, 1.27) | 1.05 (0.85, 1.29) | 0.95 (0.77, 1.18) | 0.96 (0.79, 1.18) |
| Ovulation (0-5 days before sample) | |  |  |  |  |  |
| PM_2.5_ (5 μg/m^3^) | 1.10 (0.88, 1.37) | 1.10 (0.95, 1.28) | 0.89 (0.63, 1.26) | 1.04 (0.86, 1.24) | 1.09 (0.84, 1.43) | 1.09 (0.90, 1.32) |
| NO_2_ (5 ppb) | 0.92 (0.69, 1.21) | 1.09 (0.95, 1.26) | 1.04 (0.68, 1.60) | 1.05 (0.87, 1.26) | 0.91 (0.65, 1.29) | 1.08 (0.91, 1.29) |
| O_3_ (5 ppb) | 0.85 (0.71, 1.01) | 0.88 (0.79, 0.99) | 0.92 (0.72, 1.17) | 0.95 (0.81, 1.12) | 0.86 (0.69, 1.08) | 0.90 (0.78, 1.04) |
| SO_2_ (0.5 ppb) | 0.92 (0.77, 1.09) | 0.96 (0.82, 1.13) | 1.00 (0.77, 1.32) | 1.02 (0.81, 1.27) | 0.93 (0.75, 1.16) | 0.97 (0.80, 1.18) |
| Pre-implantation (1-5 days after sample) | | |  |  |  |  |
| PM_2.5_ (5 μg/m^3^) | 1.02 (0.83, 1.26) | 1.04 (0.89, 1.21) | 0.90 (0.66, 1.22) | 0.93 (0.74, 1.18) | 1.09 (0.85, 1.39) | 1.07 (0.88, 1.28) |
| NO_2_ (5 ppb) | 1.00 (0.76, 1.32) | 1.05 (0.90, 1.21) | 0.99 (0.72, 1.36) | 1.03 (0.86, 1.25) | 0.97 (0.69, 1.37) | 1.02 (0.85, 1.23) |
| O_3_ (5 ppb) | 0.95 (0.80, 1.13) | 0.95 (0.85, 1.07) | 0.89 (0.71, 1.11) | 0.94 (0.81, 1.09) | 0.97 (0.79, 1.20) | 0.98 (0.85, 1.13) |
| SO_2_ (0.5 ppb) | 0.95 (0.81, 1.12) | 0.98 (0.84, 1.13) | 1.03 (0.82, 1.31) | 1.03 (0.84, 1.25) | 0.94 (0.76, 1.16) | 0.96 (0.79, 1.16) |
| Implantation (6-10 days after sample) | |  |  |  |  |  |
| PM_2.5_ (5 μg/m^3^) | 1.09 (0.88, 1.35) | 1.06 (0.90, 1.26) | 1.11 (0.90, 1.37) | 1.11 (0.94, 1.30) | 0.98 (0.72, 1.33) | 0.93 (0.72, 1.20) |
| NO_2_ (5 ppb) | 0.91 (0.69, 1.21) | 1.04 (0.90, 1.20) | 1.05 (0.76, 1.46) | 1.09 (0.92, 1.29) | 0.84 (0.58, 1.20) | 0.97 (0.80, 1.18) |
| O_3_ (5 ppb) | 0.89 (0.75, 1.05) | 0.93 (0.84, 1.04) | 1.01 (0.81, 1.26) | 1.00 (0.87, 1.15) | 0.82 (0.67, 1.01) | 0.93 (0.81, 1.06) |
| SO_2_ (0.5 ppb) | 0.91 (0.76, 1.08) | 0.93 (0.79, 1.09) | 1.06 (0.85, 1.33) | 1.11 (0.90, 1.37) | 0.89 (0.70, 1.12) | 0.88 (0.71, 1.09) |
| Post-implantation (11-14 days after sample) | | |  |  |  |  |
| PM_2.5_ (5 μg/m^3^) | 1.02 (0.84, 1.24) | 1.09 (0.94, 1.26) | 1.20 (1.06, 1.35) | 1.16 (1.04, 1.31) | 0.78 (0.57, 1.08) | 0.89 (0.70, 1.15) |
| NO_2_ (5 ppb) | 1.16 (0.89, 1.51) | 1.09 (0.95, 1.26) | 0.92 (0.68, 1.24) | 1.08 (0.92, 1.27) | 1.31 (0.94, 1.84) | 1.04 (0.87, 1.24) |
| O_3_ (5 ppb) | 1.06 (0.90, 1.24) | 0.98 (0.88, 1.08) | 0.91 (0.74, 1.12) | 0.94 (0.83, 1.07) | 1.11 (0.91, 1.35) | 1.01 (0.88, 1.14) |
| SO_2_ (0.5 ppb) | 0.97 (0.83, 1.13) | 1.01 (0.88, 1.16) | 1.03 (0.87, 1.24) | 1.05 (0.88, 1.25) | 0.96 (0.79, 1.17) | 0.99 (0.83, 1.18) |

PM_2.5_: Particulate matter <2.5 microns; NO_2_: Nitrogen dioxide; O_3_: Ozone; SO_2_: Sulfur dioxide. All models adjust for male age, season, income, and a baseline semen quality parameter falling below the WHO 5^th^ percentile threshold. Multipollutant models additionally adjust for co-pollutants PM_2.5_, NO_2_, O_3_, and SO_2_.

^a^Inverse probability weighting was used for multi-pollutant and single pregnancy loss models.

Table S9. Association of air pollution with pregnancy, pregnancy loss^a^, and live birth in intrauterine insemination (IUI) cycles restricted to first IUI cycle of follow-up: multipollutant and single-pollutant models

|  | Pregnancy  (n=568) | | Pregnancy loss  (n=72) | | Live birth  (n=568) | |
| --- | --- | --- | --- | --- | --- | --- |
|  | Multipollutant model | Single-pollutant model | Multipollutant model | Single-pollutant model | Multipollutant model | Single-pollutant model |
|  | RR (95% CI) | RR (95% CI) | RR (95% CI) | RR (95% CI) | RR (95% CI) | RR (95% CI) |
| Spermatogenesis (0-74 days before sample) | |  |  |  |  |  |
| PM_2.5_ (5 μg/m^3^) | 1.33 (0.89, 1.98) | 1.24 (0.91, 1.69) | 0.66 (0.24, 1.85) | 0.93 (0.51, 1.70) | 1.32 (0.84, 2.06) | 1.25 (0.87, 1.78) |
| NO_2_ (5 ppb) | 0.59 (0.39, 0.89) | 1.11 (0.91, 1.36) | 1.35 (0.60, 3.03) | 0.97 (0.69, 1.35) | 0.57 (0.35, 0.93) | 1.13 (0.87, 1.46) |
| O_3_ (5 ppb) | 0.61 (0.44, 0.84) | 0.81 (0.69, 0.96) | 1.32 (0.87, 2.01) | 1.10 (0.84, 1.46) | 0.57 (0.39, 0.85) | 0.79 (0.64, 0.97) |
| SO_2_ (0.5 ppb) | 1.06 (0.88, 1.28) | 1.05 (0.88, 1.25) | 1.09 (0.80, 1.49) | 1.04 (0.81, 1.33) | 1.06 (0.83, 1.34) | 1.04 (0.83, 1.32) |
| Mitosis (59-74 days before sample) | |  |  |  |  |  |
| PM_2.5_ (5 μg/m^3^) | 1.33 (1.04, 1.70) | 1.13 (0.94, 1.36) | 0.57 (0.27, 1.18) | 0.74 (0.51, 1.07) | 1.40 (1.05, 1.87) | 1.21 (0.97, 1.50) |
| NO_2_ (5 ppb) | 0.59 (0.42, 0.83) | 1.06 (0.91, 1.24) | 1.32 (0.59, 2.98) | 0.84 (0.63, 1.13) | 0.58 (0.39, 0.86) | 1.11 (0.92, 1.34) |
| O_3_ (5 ppb) | 0.71 (0.57, 0.88) | 0.89 (0.80, 0.99) | 1.29 (0.90, 1.83) | 1.13 (0.94, 1.35) | 0.68 (0.53, 0.88) | 0.85 (0.74, 0.98) |
| SO_2_ (0.5 ppb) | 1.11 (0.95, 1.29) | 1.07 (0.92, 1.24) | 1.12 (0.82, 1.54) | 1.00 (0.78, 1.28) | 1.10 (0.93, 1.32) | 1.08 (0.92, 1.28) |
| Meiosis I+II (33-58 days before sample) | |  |  |  |  |  |
| PM_2.5_ (5 μg/m^3^) | 1.31 (0.96, 1.79) | 1.16 (0.91, 1.49) | 1.21 (0.68, 2.15) | 1.19 (0.84, 1.68) | 1.12 (0.77, 1.61) | 1.06 (0.78, 1.45) |
| NO_2_ (5 ppb) | 0.65 (0.43, 0.97) | 1.10 (0.91, 1.31) | 0.89 (0.50, 1.56) | 0.96 (0.72, 1.28) | 0.67 (0.41, 1.11) | 1.11 (0.89, 1.40) |
| O_3_ (5 ppb) | 0.67 (0.50, 0.91) | 0.85 (0.73, 0.98) | 1.16 (0.86, 1.57) | 1.12 (0.89, 1.40) | 0.64 (0.44, 0.93) | 0.82 (0.68, 0.98) |
| SO_2_ (0.5 ppb) | 1.04 (0.87, 1.25) | 1.04 (0.87, 1.24) | 1.11 (0.81, 1.50) | 1.04 (0.80, 1.36) | 1.03 (0.82, 1.30) | 1.02 (0.81, 1.29) |
| Spermiogenesis (11-32 days before sample) | |  |  |  |  |  |
| PM_2.5_ (5 μg/m^3^) | 0.99 (0.70, 1.40) | 1.07 (0.83, 1.38) | 0.77 (0.39, 1.52) | 0.95 (0.58, 1.57) | 1.00 (0.65, 1.54) | 1.06 (0.80, 1.42) |
| NO_2_ (5 ppb) | 0.88 (0.62, 1.25) | 1.10 (0.92, 1.32) | 1.23 (0.63, 2.41) | 1.03 (0.75, 1.41) | 0.84 (0.55, 1.27) | 1.08 (0.87, 1.35) |
| O_3_ (5 ppb) | 0.78 (0.59, 1.04) | 0.86 (0.72, 1.02) | 1.20 (0.72, 2.00) | 1.03 (0.75, 1.41) | 0.76 (0.54, 1.06) | 0.86 (0.70, 1.05) |
| SO_2_ (0.5 ppb) | 1.03 (0.86, 1.24) | 1.05 (0.88, 1.24) | 1.05 (0.82, 1.36) | 1.03 (0.83, 1.28) | 1.02 (0.80, 1.32) | 1.03 (0.81, 1.31) |
| Spermiation (0-10 days before sample) | |  |  |  |  |  |
| PM_2.5_ (5 μg/m^3^) | 1.07 (0.85, 1.36) | 1.09 (0.87, 1.36) | 0.55 (0.29, 1.01) | 0.89 (0.57, 1.38) | 1.16 (0.90, 1.49) | 1.13 (0.87, 1.46) |
| NO_2_ (5 ppb) | 0.81 (0.61, 1.09) | 1.07 (0.88, 1.31) | 1.61 (0.82, 3.19) | 1.15 (0.87, 1.52) | 0.79 (0.55, 1.13) | 1.04 (0.80, 1.35) |
| O_3_ (5 ppb) | 0.77 (0.63, 0.94) | 0.85 (0.73, 0.98) | 1.00 (0.69, 1.44) | 0.88 (0.65, 1.18) | 0.80 (0.62, 1.03) | 0.88 (0.73, 1.06) |
| SO_2_ (0.5 ppb) | 1.01 (0.83, 1.22) | 1.02 (0.86, 1.21) | 1.01 (0.73, 1.38) | 1.08 (0.84, 1.38) | 0.99 (0.77, 1.28) | 0.99 (0.78, 1.25) |
| Ovulation (0-5 days before sample) | |  |  |  |  |  |
| PM_2.5_ (5 μg/m^3^) | 1.20 (0.93, 1.54) | 1.15 (0.95, 1.40) | 0.41 (0.16, 1.05) | 0.71 (0.42, 1.19) | 1.36 (1.05, 1.74) | 1.24 (1.02, 1.51) |
| NO_2_ (5 ppb) | 0.76 (0.58, 1.00) | 1.10 (0.92, 1.33) | 1.77 (0.91, 3.44) | 1.04 (0.80, 1.35) | 0.72 (0.53, 0.97) | 1.11 (0.87, 1.41) |
| O_3_ (5 ppb) | 0.74 (0.61, 0.88) | 0.82 (0.72, 0.94) | 1.06 (0.74, 1.53) | 0.96 (0.72, 1.29) | 0.74 (0.59, 0.93) | 0.82 (0.70, 0.97) |
| SO_2_ (0.5 ppb) | 1.00 (0.82, 1.22) | 1.02 (0.85, 1.22) | 1.00 (0.73, 1.37) | 1.03 (0.80, 1.33) | 0.99 (0.76, 1.28) | 1.01 (0.80, 1.28) |
| Pre-implantation (1-5 days after sample) | |  |  |  |  |  |
| PM_2.5_ (5 μg/m^3^) | 1.10 (0.89, 1.35) | 1.09 (0.93, 1.28) | 0.81 (0.55, 1.18) | 0.83 (0.64, 1.08) | 1.19 (0.95, 1.49) | 1.14 (0.94, 1.37) |
| NO_2_ (5 ppb) | 0.89 (0.67, 1.17) | 1.07 (0.91, 1.27) | 0.98 (0.58, 1.66) | 1.05 (0.79, 1.40) | 0.90 (0.65, 1.25) | 1.05 (0.84, 1.31) |
| O_3_ (5 ppb) | 0.88 (0.73, 1.06) | 0.91 (0.80, 1.04) | 0.80 (0.59, 1.09) | 0.86 (0.68, 1.09) | 0.96 (0.76, 1.21) | 0.96 (0.82, 1.13) |
| SO_2_ (0.5 ppb) | 1.02 (0.88, 1.20) | 1.04 (0.90, 1.21) | 1.06 (0.80, 1.40) | 1.07 (0.82, 1.41) | 0.99 (0.82, 1.21) | 1.01 (0.84, 1.22) |
| Implantation (6-10 days after sample) | |  |  |  |  |  |
| PM_2.5_ (5 μg/m^3^) | 1.11 (0.88, 1.40) | 1.10 (0.92, 1.31) | 1.20 (0.90, 1.60) | 1.17 (0.95, 1.44) | 1.02 (0.77, 1.34) | 0.98 (0.77, 1.26) |
| NO_2_ (5 ppb) | 0.87 (0.62, 1.21) | 1.10 (0.91, 1.33) | 0.91 (0.59, 1.40) | 1.19 (0.96, 1.48) | 0.85 (0.55, 1.32) | 1.00 (0.77, 1.30) |
| O_3_ (5 ppb) | 0.84 (0.69, 1.01) | 0.88 (0.77, 1.01) | 0.86 (0.63, 1.16) | 0.89 (0.72, 1.11) | 0.85 (0.67, 1.09) | 0.93 (0.78, 1.09) |
| SO_2_ (0.5 ppb) | 0.99 (0.82, 1.20) | 1.01 (0.85, 1.20) | 1.06 (0.79, 1.44) | 1.15 (0.89, 1.47) | 0.96 (0.76, 1.22) | 0.96 (0.76, 1.20) |
| Post-implantation (11-14 days after sample) | |  |  |  |  |  |
| PM_2.5_ (5 μg/m^3^) | 1.10 (0.89, 1.36) | 1.15 (0.97, 1.35) | 1.21 (1.02, 1.44) | 1.20 (1.04, 1.39) | 0.91 (0.7, 1.19) | 0.97 (0.75, 1.24) |
| NO_2_ (5 ppb) | 1.07 (0.78, 1.47) | 1.13 (0.93, 1.38) | 1.00 (0.73, 1.37) | 1.16 (0.93, 1.44) | 1.12 (0.74, 1.68) | 1.04 (0.80, 1.34) |
| O_3_ (5 ppb) | 0.99 (0.83, 1.19) | 0.94 (0.82, 1.08) | 0.89 (0.67, 1.19) | 0.91 (0.73, 1.14) | 1.03 (0.82, 1.30) | 0.99 (0.84, 1.17) |
| SO_2_ (0.5 ppb) | 1.00 (0.83, 1.21) | 1.04 (0.88, 1.23) | 1.02 (0.79, 1.32) | 1.07 (0.83, 1.37) | 0.99 (0.77, 1.28) | 1.01 (0.81, 1.27) |

PM_2.5_: Particulate matter <2.5 microns; NO_2_: Nitrogen dioxide; O_3_: Ozone; SO_2_: Sulfur dioxide. All models adjust for male age, season, and income. Multipollutant models additionally adjust for co-pollutants PM_2.5_, NO_2_, O_3_, and SO_2_.

^a^Inverse probability weighting was used for multi-pollutant and single pregnancy loss models.

Table S10. Association of air pollution with pregnancy, pregnancy loss^a^, and live birth in intrauterine insemination (IUI) cycles additionally co-adjusted for windows of exposure during spermatogenesis: multipollutant and single-pollutant models.

|  | Pregnancy  (n=1,176) | | Pregnancy loss  (n=127) | | Live birth  (n=1,176) | |
| --- | --- | --- | --- | --- | --- | --- |
|  | Multipollutant | Single-pollutant | Multipollutant | Single-pollutant | Multipollutant | Single-pollutant |
|  | RR (95% CI) | RR (95% CI) | RR (95% CI) | RR (95% CI) | RR (95% CI) | RR (95% CI) |
| Mitosis (59-74 days before sample) | |  |  |  |  |  |
| PM_2.5_ (5 μg/m^3^) | 1.35 (1.01, 1.80) | 1.14 (0.93, 1.39) | 0.89 (0.47, 1.66) | 0.79 (0.53, 1.17) | 1.42 (1.00, 2.01) | 1.24 (0.99, 1.57) |
| NO_2_ (5 ppb) | 0.66 (0.43, 1.03) | 0.97 (0.74, 1.25) | 0.88 (0.39, 2.01) | 0.93 (0.60, 1.44) | 0.70 (0.41, 1.20) | 1.00 (0.74, 1.35) |
| O_3_ (5 ppb) | 0.79 (0.59, 1.08) | 0.94 (0.73, 1.20) | 0.72 (0.47, 1.10) | 0.79 (0.58, 1.07) | 0.95 (0.66, 1.37) | 1.04 (0.77, 1.40) |
| Meiosis I+II (33-58 days before sample) | |  |  |  |  |  |
| PM_2.5_ (5 μg/m^3^) | 1.06 (0.70, 1.61) | 1.09 (0.81, 1.45) | 1.73 (0.97, 3.10) | 1.31 (0.81, 2.10) | 0.80 (0.46, 1.37) | 0.98 (0.68, 1.41) |
| NO_2_ (5 ppb) | 0.95 (0.53, 1.72) | 1.18 (0.80, 1.74) | 0.42 (0.19, 0.97) | 0.88 (0.51, 1.49) | 1.31 (0.63, 2.75) | 1.23 (0.78, 1.94) |
| O_3_ (5 ppb) | 0.85 (0.55, 1.31) | 0.86 (0.59, 1.25) | 0.97 (0.49, 1.93) | 1.40 (0.90, 2.17) | 0.79 (0.46, 1.36) | 0.76 (0.48, 1.20) |
| Spermiogenesis (11-32 days before sample) | | |  |  |  |  |
| PM_2.5_ (5 μg/m^3^) | 0.82 (0.52, 1.30) | 0.94 (0.69, 1.28) | 0.73 (0.38, 1.40) | 0.76 (0.50, 1.17) | 0.96 (0.56, 1.66) | 1.05 (0.73, 1.53) |
| NO_2_ (5 ppb) | 1.23 (0.68, 2.23) | 0.90 (0.62, 1.31) | 1.45 (0.55, 3.83) | 0.84 (0.53, 1.33) | 1.08 (0.52, 2.26) | 0.99 (0.63, 1.57) |
| O_3_ (5 ppb) | 1.34 (0.91, 1.99) | 1.31 (0.94, 1.82) | 1.75 (0.86, 3.56) | 1.22 (0.79, 1.87) | 1.16 (0.72, 1.89) | 1.18 (0.78, 1.76) |
| Spermiation (0-10 days before sample) | |  |  |  |  |  |
| PM_2.5_ (5 μg/m^3^) | 1.11 (0.81, 1.50) | 1.12 (0.92, 1.36) | 0.74 (0.45, 1.22) | 1.15 (0.97, 1.35) | 1.11 (0.76, 1.62) | 1.01 (0.77, 1.31) |
| NO_2_ (5 ppb) | 0.84 (0.54, 1.32) | 1.09 (0.84, 1.40) | 1.68 (0.82, 3.44) | 1.45 (1.10, 1.90) | 0.74 (0.43, 1.30) | 0.91 (0.66, 1.27) |
| O_3_ (5 ppb) | 0.74 (0.55, 0.99) | 0.79 (0.62, 0.99) | 0.69 (0.45, 1.06) | 0.69 (0.50, 0.94) | 0.82 (0.58, 1.18) | 0.92 (0.69, 1.23) |

PM_2.5_: Particulate matter <2.5 microns; NO_2_: Nitrogen dioxide; O_3_: Ozone; SO_2_: Sulfur dioxide. All models adjust for male age, season, and income. Multipollutant models additionally adjust for co-pollutants PM_2.5_, NO_2_, O_3_, and SO_2_.

^a^Inverse probability weighting was used for multi-pollutant and single pregnancy loss models.

Table S11. Association of air pollution and pregnancy in intrauterine insemination (IUI) cycles by treatment assignment (multipollutant models) (n=1,176).

|  | Pregnancy by treatment assignment | | |
| --- | --- | --- | --- |
|  | Multipollutant | | |
|  | Placebo | Folic acid + zinc | RERI |
|  | RR (95% CI) | RR (95% CI) | Estimate (95% CI) |
| Spermatogenesis (0-74 days before sample) | |  |  |
| PM_2.5_ (5 μg/m^3^) | 1.10 (0.65, 1.87) | 1.43 (0.88, 2.31) | -0.057 (-0.151, 0.040) |
| NO_2_ (5 ppb) | 0.66 (0.40, 1.09) | 0.74 (0.45, 1.23) | 0.001 (-0.119, 0.098) |
| O_3_ (5 ppb) | 0.77 (0.56, 1.06) | 0.65 (0.47, 0.91) | 0.015 (-0.052, 0.069) |
| SO_2_ (0.5 ppb) | 0.95 (0.71, 1.26) | 0.99 (0.79, 1.24) | -0.005 (-0.044, 0.031) |
| Mitosis (59-74 days before sample) |  |  |  |
| PM_2.5_ (5 μg/m^3^) | 1.33 (0.98, 1.81) | 1.24 (0.85, 1.82) | -0.020 (-0.084, 0.040) |
| NO_2_ (5 ppb) | 0.69 (0.45, 1.06) | 0.71 (0.45, 1.11) | 0.032 (-0.052, 0.108) |
| O_3_ (5 ppb) | 0.84 (0.66, 1.06) | 0.70 (0.55, 0.90) | 0.030 (-0.016, 0.069) |
| SO_2_ (0.5 ppb) | 1.02 (0.82, 1.28) | 0.98 (0.79, 1.23) | 0.011 (-0.023, 0.045) |
| Meiosis I+II (33-58 days before sample) | |  |  |
| PM_2.5_ (5 μg/m^3^) | 0.99 (0.63, 1.55) | 1.30 (0.91, 1.85) | -0.046 (-0.119, 0.032) |
| NO_2_ (5 ppb) | 0.73 (0.46, 1.15) | 0.86 (0.56, 1.31) | 0.002 (-0.103, 0.088) |
| O_3_ (5 ppb) | 0.81 (0.61, 1.07) | 0.73 (0.56, 0.97) | 0.015 (-0.039, 0.060) |
| SO_2_ (0.5 ppb) | 0.91 (0.68, 1.23) | 0.99 (0.78, 1.25) | -0.016 (-0.054, 0.019) |
| Spermiogenesis (11-32 days before sample) | |  |  |
| PM_2.5_ (5 μg/m^3^) | 0.97 (0.61, 1.53) | 1.07 (0.71, 1.59) | -0.016 (-0.079, 0.048) |
| NO_2_ (5 ppb) | 0.81 (0.51, 1.29) | 0.98 (0.63, 1.50) | -0.008 (-0.098, 0.062) |
| O_3_ (5 ppb) | 0.93 (0.70, 1.24) | 0.77 (0.57, 1.03) | 0.018 (-0.032, 0.054) |
| SO_2_ (0.5 ppb) | 0.89 (0.66, 1.20) | 0.99 (0.81, 1.21) | -0.010 (-0.046, 0.029) |
| Spermiation (0-10 days before sample) | |  |  |
| PM_2.5_ (5 μg/m^3^) | 1.05 (0.73, 1.50) | 1.08 (0.81, 1.45) | -0.011 (-0.075, 0.057) |
| NO_2_ (5 ppb) | 0.78 (0.52, 1.19) | 0.96 (0.66, 1.39) | -0.031 (-0.121, 0.037) |
| O_3_ (5 ppb) | 0.90 (0.71, 1.13) | 0.77 (0.60, 0.98) | 0.001 (-0.044, 0.033) |
| SO_2_ (0.5 ppb) | 0.91 (0.68, 1.22) | 0.96 (0.78, 1.19) | -0.004 (-0.051, 0.041) |
| Ovulation (0-5 days before sample) |  |  |  |
| PM_2.5_ (5 μg/m^3^) | 1.25 (0.92, 1.69) | 0.99 (0.72, 1.35) | 0.041 (-0.025, 0.104) |
| NO_2_ (5 ppb) | 0.74 (0.51, 1.08) | 1.05 (0.69, 1.58) | -0.064 (-0.142, 0.011) |
| O_3_ (5 ppb) | 0.89 (0.73, 1.10) | 0.79 (0.62, 1.01) | -0.011 (-0.052, 0.023) |
| SO_2_ (0.5 ppb) | 0.86 (0.63, 1.16) | 0.97 (0.78, 1.20) | -0.015 (-0.055, 0.024) |
| Pre-implantation (1-5 days after sample) | |  |  |
| PM_2.5_ (5 μg/m^3^) | 1.13 (0.85, 1.49) | 1.02 (0.79, 1.32) | 0.042 (-0.010, 0.104) |
| NO_2_ (5 ppb) | 0.74 (0.50, 1.08) | 1.10 (0.74, 1.62) | -0.055 (-0.152, 0.023) |
| O_3_ (5 ppb) | 0.96 (0.79, 1.18) | 0.86 (0.68, 1.10) | 0.004 (-0.041, 0.038) |
| SO_2_ (0.5 ppb) | 0.94 (0.71, 1.24) | 0.98 (0.81, 1.19) | -0.003 (-0.045, 0.037) |
| Implantation (6-10 days after sample) |  |  |  |
| PM_2.5_ (5 μg/m^3^) | 1.35 (0.96, 1.90) | 0.98 (0.74, 1.29) | 0.059 (-0.003, 0.129) |
| NO_2_ (5 ppb) | 0.67 (0.44, 1.00) | 1.05 (0.70, 1.58) | -0.051 (-0.142, 0.021) |
| O_3_ (5 ppb) | 0.90 (0.74, 1.10) | 0.83 (0.65, 1.06) | 0.001 (-0.048, 0.036) |
| SO_2_ (0.5 ppb) | 0.92 (0.70, 1.22) | 0.92 (0.74, 1.15) | 0.005 (-0.028, 0.038) |
| Post-implantation (11-14 days after sample) | |  |  |
| PM_2.5_ (5 μg/m^3^) | 1.21 (0.87, 1.68) | 0.95 (0.74, 1.22) | 0.040 (-0.012, 0.093) |
| NO_2_ (5 ppb) | 0.83 (0.56, 1.25) | 1.34 (0.94, 1.92) | -0.066 (-0.140, -0.001) |
| O_3_ (5 ppb) | 1.07 (0.87, 1.31) | 0.99 (0.79, 1.24) | -0.004 (-0.038, 0.024) |
| SO_2_ (0.5 ppb) | 1.02 (0.83, 1.26) | 0.94 (0.75, 1.17) | 0.018 (-0.025, 0.058) |

PM_2.5_: Particulate matter <2.5 microns; NO_2_: Nitrogen dioxide; O_3_: Ozone; SO_2_: Sulfur dioxide. All models adjust for male age, season, and income. Multipollutant models additionally adjust for co-pollutants PM_2.5_, NO_2_, O_3_, and SO_2_.

Table S12. Association of air pollution and live birth in intrauterine insemination (IUI) cycles by treatment assignment (n=1,176).

|  | Live birth by treatment assignment | | |
| --- | --- | --- | --- |
|  | Multipollutant | | |
|  | Placebo | Folic acid + zinc | RERI |
|  | RR (95% CI) | RR (95% CI) | Estimate (95% CI) |
| Spermatogenesis (0-74 days before sample) |  |  |  |
| PM_2.5_ (5 μg/m^3^) | 1.03 (0.54, 1.98) | 1.34 (0.72, 2.49) | -0.020 (-0.074, 0.033) |
| NO_2_ (5 ppb) | 0.76 (0.41, 1.40) | 0.83 (0.44, 1.58) | 0.019 (-0.065, 0.083) |
| O_3_ (5 ppb) | 0.82 (0.55, 1.21) | 0.70 (0.46, 1.07) | 0.017 (-0.025, 0.049) |
| SO_2_ (0.5 ppb) | 0.98 (0.71, 1.36) | 0.97 (0.72, 1.31) | -0.004 (-0.036, 0.028) |
| Mitosis (59-74 days before sample) |  |  |  |
| PM_2.5_ (5 μg/m^3^) | 1.26 (0.87, 1.84) | 1.40 (0.88, 2.22) | -0.042 (-0.084, -0.003) |
| NO_2_ (5 ppb) | 0.82 (0.48, 1.39) | 0.75 (0.43, 1.29) | 0.048 (-0.009, 0.093) |
| O_3_ (5 ppb) | 0.90 (0.67, 1.21) | 0.75 (0.55, 1.02) | 0.024 (-0.007, 0.049) |
| SO_2_ (0.5 ppb) | 1.05 (0.83, 1.33) | 0.96 (0.71, 1.29) | 0.005 (-0.025, 0.034) |
| Meiosis I+II (33-58 days before sample) |  |  |  |
| PM_2.5_ (5 μg/m^3^) | 0.81 (0.45, 1.47) | 1.19 (0.74, 1.91) | -0.017 (-0.068, 0.034) |
| NO_2_ (5 ppb) | 0.83 (0.46, 1.49) | 1.03 (0.59, 1.80) | -0.013 (-0.090, 0.050) |
| O_3_ (5 ppb) | 0.83 (0.59, 1.19) | 0.81 (0.57, 1.16) | 0.000 (-0.043, 0.033) |
| SO_2_ (0.5 ppb) | 0.96 (0.68, 1.35) | 0.95 (0.69, 1.30) | -0.007 (-0.042, 0.026) |
| Spermiogenesis (11-32 days before sample) |  |  |  |
| PM_2.5_ (5 μg/m^3^) | 0.94 (0.54, 1.63) | 1.09 (0.66, 1.81) | -0.017 (-0.057, 0.023) |
| NO_2_ (5 ppb) | 0.89 (0.51, 1.56) | 0.94 (0.55, 1.63) | 0.027 (-0.029, 0.079) |
| O_3_ (5 ppb) | 0.96 (0.68, 1.36) | 0.77 (0.53, 1.11) | 0.021 (-0.010, 0.050) |
| SO_2_ (0.5 ppb) | 0.90 (0.63, 1.28) | 1.02 (0.79, 1.31) | -0.017 (-0.046, 0.014) |
| Spermiation (0-10 days before sample) |  |  |  |
| PM_2.5_ (5 μg/m^3^) | 1.16 (0.79, 1.71) | 0.87 (0.55, 1.36) | 0.041 (-0.001, 0.088) |
| NO_2_ (5 ppb) | 0.75 (0.46, 1.23) | 1.04 (0.61, 1.76) | -0.031 (-0.090, 0.014) |
| O_3_ (5 ppb) | 0.92 (0.70, 1.21) | 0.80 (0.58, 1.10) | 0.002 (-0.027, 0.027) |
| SO_2_ (0.5 ppb) | 0.98 (0.71, 1.36) | 0.95 (0.71, 1.27) | 0.012 (-0.021, 0.050) |
| Ovulation (0-5 days before sample) |  |  |  |
| PM_2.5_ (5 μg/m^3^) | 1.33 (0.94, 1.86) | 0.91 (0.60, 1.37) | 0.062 (0.011, 0.119) |
| NO_2_ (5 ppb) | 0.69 (0.44, 1.08) | 1.15 (0.68, 1.95) | -0.067 (-0.133, -0.009) |
| O_3_ (5 ppb) | 0.88 (0.69, 1.13) | 0.85 (0.62, 1.15) | -0.016 (-0.051, 0.013) |
| SO_2_ (0.5 ppb) | 0.92 (0.66, 1.30) | 0.97 (0.73, 1.27) | 0.000 (-0.024, 0.025) |
| Pre-implantation (1-5 days after sample) |  |  |  |
| PM_2.5_ (5 μg/m^3^) | 1.09 (0.77, 1.55) | 1.17 (0.87, 1.56) | 0.037 (-0.008, 0.089) |
| NO_2_ (5 ppb) | 0.70 (0.44, 1.12) | 1.07 (0.66, 1.74) | -0.049 (-0.119, 0.015) |
| O_3_ (5 ppb) | 0.96 (0.75, 1.23) | 0.92 (0.68, 1.24) | 0.000 (-0.034, 0.026) |
| SO_2_ (0.5 ppb) | 1.02 (0.75, 1.39) | 0.92 (0.70, 1.22) | 0.021 (-0.022, 0.067) |
| Implantation (6-10 days after sample) |  |  |  |
| PM_2.5_ (5 μg/m^3^) | 1.24 (0.80, 1.91) | 0.85 (0.56, 1.29) | 0.064 (0.021, 0.113) |
| NO_2_ (5 ppb) | 0.58 (0.35, 0.96) | 1.08 (0.64, 1.83) | -0.039 (-0.102, 0.010) |
| O_3_ (5 ppb) | 0.80 (0.63, 1.02) | 0.84 (0.62, 1.14) | -0.004 (-0.037, 0.022) |
| SO_2_ (0.5 ppb) | 0.99 (0.72, 1.35) | 0.84 (0.60, 1.17) | 0.011 (-0.016, 0.038) |
| Post-implantation (11-14 days after sample) |  |  |  |
| PM_2.5_ (5 μg/m^3^) | 0.94 (0.57, 1.56) | 0.70 (0.47, 1.05) | 0.030 (-0.005, 0.069) |
| NO_2_ (5 ppb) | 0.85 (0.52, 1.40) | 1.72 (1.09, 2.71) | -0.028 (-0.082, 0.018) |
| O_3_ (5 ppb) | 1.05 (0.82, 1.35) | 1.11 (0.84, 1.46) | 0.006 (-0.015, 0.023) |
| SO_2_ (0.5 ppb) | 1.08 (0.86, 1.37) | 0.84 (0.60, 1.17) | 0.020 (-0.011, 0.059) |

PM_2.5_: Particulate matter <2.5 microns; NO_2_: Nitrogen dioxide; O_3_: Ozone; SO_2_: Sulfur dioxide. All models adjust for male age, season, and income. Multipollutant models additionally adjust for co-pollutants PM_2.5_, NO_2_, O_3_, and SO_2_.

Table S13. Association of air pollution and pregnancy in intrauterine insemination (IUI) cycles by history of male partner infertility diagnosis (n=1,176).

|  | Pregnancy by history of male partner infertility diagnosis | | |
| --- | --- | --- | --- |
|  | Multipollutant | | |
|  | Yes | No/missing | RERI |
|  | RR (95% CI) | RR (95% CI) | Estimate (95% CI) |
| Spermatogenesis (0-74 days before sample) | |  |  |
| PM_2.5_ (5 μg/m^3^) | 0.77 (0.28, 2.11) | 1.42 (0.97, 2.06) | -0.095 (-0.202, 0.021) |
| NO_2_ (5 ppb) | 1.07 (0.39, 2.94) | 0.73 (0.49, 1.07) | 0.036 (-0.161, 0.144) |
| O_3_ (5 ppb) | 0.81 (0.46, 1.43) | 0.72 (0.55, 0.94) | 0.000 (-0.125, 0.063) |
| SO_2_ (0.5 ppb) | 1.00 (0.67, 1.50) | 0.94 (0.77, 1.15) | -0.002 (-0.044, 0.039) |
| Mitosis (59-74 days before sample) |  |  |  |
| PM_2.5_ (5 μg/m^3^) | 1.00 (0.51, 1.96) | 1.35 (1.04, 1.75) | -0.038 (-0.112, 0.068) |
| NO_2_ (5 ppb) | 1.13 (0.48, 2.69) | 0.66 (0.47, 0.93) | 0.042 (-0.113, 0.133) |
| O_3_ (5 ppb) | 0.95 (0.60, 1.52) | 0.76 (0.63, 0.92) | 0.013 (-0.067, 0.060) |
| SO_2_ (0.5 ppb) | 0.99 (0.70, 1.41) | 1.00 (0.84, 1.19) | 0.009 (-0.024, 0.053) |
| Meiosis I+II (33-58 days before sample) | |  |  |
| PM_2.5_ (5 μg/m^3^) | 0.69 (0.29, 1.63) | 1.31 (0.98, 1.77) | -0.069 (-0.165, 0.053) |
| NO_2_ (5 ppb) | 1.13 (0.45, 2.86) | 0.79 (0.56, 1.12) | 0.018 (-0.144, 0.115) |
| O_3_ (5 ppb) | 0.83 (0.49, 1.39) | 0.77 (0.61, 0.97) | -0.008 (-0.116, 0.050) |
| SO_2_ (0.5 ppb) | 1.04 (0.70, 1.54) | 0.91 (0.74, 1.12) | 0.011 (-0.041, 0.056) |
| Spermiogenesis (11-32 days before sample) | |  |  |
| PM_2.5_ (5 μg/m^3^) | 0.80 (0.37, 1.74) | 1.08 (0.79, 1.48) | -0.010 (-0.082, 0.076) |
| NO_2_ (5 ppb) | 1.11 (0.51, 2.41) | 0.96 (0.68, 1.36) | -0.007 (-0.134, 0.076) |
| O_3_ (5 ppb) | 0.92 (0.58, 1.46) | 0.85 (0.66, 1.08) | -0.005 (-0.084, 0.042) |
| SO_2_ (0.5 ppb) | 0.91 (0.60, 1.39) | 0.95 (0.79, 1.15) | -0.029 (-0.082, 0.011) |
| Spermiation (0-10 days before sample) | |  |  |
| PM_2.5_ (5 μg/m^3^) | 0.94 (0.55, 1.63) | 1.14 (0.88, 1.47) | -0.027 (-0.105, 0.061) |
| NO_2_ (5 ppb) | 1.00 (0.51, 1.96) | 0.91 (0.67, 1.25) | -0.011 (-0.113, 0.064) |
| O_3_ (5 ppb) | 0.88 (0.59, 1.30) | 0.83 (0.68, 1.02) | -0.003 (-0.055, 0.030) |
| SO_2_ (0.5 ppb) | 1.05 (0.75, 1.47) | 0.91 (0.74, 1.11) | 0.030 (-0.023, 0.094) |
| Ovulation (0-5 days before sample) |  |  |  |
| PM_2.5_ (5 μg/m^3^) | 1.05 (0.65, 1.70) | 1.13 (0.89, 1.45) | -0.010 (-0.079, 0.076) |
| NO_2_ (5 ppb) | 1.00 (0.50, 2.01) | 0.90 (0.66, 1.21) | -0.007 (-0.096, 0.064) |
| O_3_ (5 ppb) | 0.90 (0.61, 1.33) | 0.83 (0.69, 1.00) | 0.002 (-0.042, 0.035) |
| SO_2_ (0.5 ppb) | 1.01 (0.72, 1.42) | 0.90 (0.73, 1.10) | 0.022 (-0.022, 0.073) |
| Pre-implantation (1-5 days after sample) | |  |  |
| PM_2.5_ (5 μg/m^3^) | 1.09 (0.70, 1.69) | 1.05 (0.85, 1.31) | 0.013 (-0.042, 0.091) |
| NO_2_ (5 ppb) | 1.07 (0.55, 2.10) | 0.97 (0.71, 1.31) | 0.001 (-0.111, 0.077) |
| O_3_ (5 ppb) | 1.03 (0.71, 1.50) | 0.91 (0.76, 1.10) | 0.010 (-0.038, 0.041) |
| SO_2_ (0.5 ppb) | 1.05 (0.79, 1.39) | 0.93 (0.77, 1.13) | 0.032 (-0.009, 0.081) |
| Implantation (6-10 days after sample) | |  |  |
| PM_2.5_ (5 μg/m^3^) | 1.20 (0.65, 2.22) | 1.09 (0.87, 1.36) | 0.039 (-0.057, 0.157) |
| NO_2_ (5 ppb) | 1.04 (0.55, 1.98) | 0.87 (0.63, 1.19) | -0.009 (-0.107, 0.066) |
| O_3_ (5 ppb) | 1.01 (0.71, 1.44) | 0.85 (0.71, 1.02) | 0.014 (-0.026, 0.043) |
| SO_2_ (0.5 ppb) | 0.92 (0.66, 1.29) | 0.92 (0.75, 1.13) | 0.015 (-0.019, 0.053) |
| Post-implantation (11-14 days after sample) | |  |  |
| PM_2.5_ (5 μg/m^3^) | 1.04 (0.63, 1.71) | 1.02 (0.83, 1.26) | 0.020 (-0.035, 0.080) |
| NO_2_ (5 ppb) | 1.16 (0.63, 2.14) | 1.18 (0.88, 1.59) | -0.020 (-0.091, 0.036) |
| O_3_ (5 ppb) | 1.11 (0.78, 1.58) | 1.04 (0.87, 1.23) | 0.003 (-0.032, 0.029) |
| SO_2_ (0.5 ppb) | 1.09 (0.84, 1.40) | 0.92 (0.76, 1.12) | 0.033 (-0.010, 0.080) |

PM_2.5_: Particulate matter <2.5 microns; NO_2_: Nitrogen dioxide; O_3_: Ozone; SO_2_: Sulfur dioxide. All models adjust for male age, season, and income. Multipollutant models additionally adjust for co-pollutants PM_2.5_, NO_2_, O_3_, and SO_2_.

Table S14. Association of air pollution and live birth in intrauterine insemination (IUI) cycles by history of male partner infertility diagnosis (n=1,176).

|  | Live birth by history of male partner infertility diagnosis | | |
| --- | --- | --- | --- |
|  | Multipollutant | | |
|  | Yes | No/missing | RERI |
|  | RR (95% CI) | RR (95% CI) | Estimate (95% CI) |
| Spermatogenesis (0-74 days before sample) | |  |  |
| PM_2.5_ (5 μg/m^3^) | 1.35 (0.44, 4.14) | 1.16 (0.71, 1.89) | -0.024 (-0.094, 0.044) |
| NO_2_ (5 ppb) | 1.07 (0.32, 3.63) | 0.82 (0.50, 1.34) | 0.037 (-0.058, 0.108) |
| O_3_ (5 ppb) | 0.96 (0.48, 1.94) | 0.74 (0.53, 1.05) | 0.017 (-0.030, 0.049) |
| SO_2_ (0.5 ppb) | 1.07 (0.66, 1.72) | 0.94 (0.73, 1.21) | 0.002 (-0.026, 0.030) |
| Mitosis (59-74 days before sample) | |  |  |
| PM_2.5_ (5 μg/m^3^) | 1.27 (0.59, 2.70) | 1.32 (0.96, 1.82) | 0.004 (-0.047, 0.075) |
| NO_2_ (5 ppb) | 1.03 (0.36, 2.92) | 0.77 (0.51, 1.16) | 0.007 (-0.096, 0.070) |
| O_3_ (5 ppb) | 0.93 (0.52, 1.68) | 0.82 (0.65, 1.04) | 0.001 (-0.050, 0.032) |
| SO_2_ (0.5 ppb) | 1.07 (0.73, 1.57) | 0.99 (0.80, 1.22) | 0.013 (-0.020, 0.060) |
| Meiosis I+II (33-58 days before sample) | |  |  |
| PM_2.5_ (5 μg/m^3^) | 0.77 (0.27, 2.17) | 1.10 (0.75, 1.62) | -0.038 (-0.123, 0.037) |
| NO_2_ (5 ppb) | 1.32 (0.42, 4.14) | 0.94 (0.60, 1.46) | 0.026 (-0.045, 0.099) |
| O_3_ (5 ppb) | 0.92 (0.48, 1.75) | 0.82 (0.61, 1.10) | 0.003 (-0.037, 0.037) |
| SO_2_ (0.5 ppb) | 1.06 (0.65, 1.74) | 0.90 (0.69, 1.17) | -0.004 (-0.050, 0.032) |
| Spermiogenesis (11-32 days before sample) | |  |  |
| PM_2.5_ (5 μg/m^3^) | 1.19 (0.47, 3.00) | 0.98 (0.66, 1.47) | 0.009 (-0.036, 0.067) |
| NO_2_ (5 ppb) | 1.14 (0.43, 3.02) | 0.96 (0.62, 1.49) | 0.015 (-0.065, 0.073) |
| O_3_ (5 ppb) | 1.10 (0.62, 1.94) | 0.83 (0.61, 1.12) | 0.018 (-0.026, 0.045) |
| SO_2_ (0.5 ppb) | 0.98 (0.59, 1.62) | 0.96 (0.76, 1.21) | -0.010 (-0.041, 0.021) |
| Spermiation (0-10 days before sample) | |  |  |
| PM_2.5_ (5 μg/m^3^) | 1.27 (0.76, 2.11) | 0.92 (0.64, 1.33) | 0.014 (-0.052, 0.088) |
| NO_2_ (5 ppb) | 0.98 (0.49, 1.93) | 0.92 (0.62, 1.38) | -0.010 (-0.078, 0.054) |
| O_3_ (5 ppb) | 1.05 (0.67, 1.65) | 0.83 (0.64, 1.07) | 0.005 (-0.024, 0.030) |
| SO_2_ (0.5 ppb) | 1.16 (0.78, 1.73) | 0.91 (0.70, 1.18) | 0.024 (-0.014, 0.068) |
| Ovulation (0-5 days before sample) | |  |  |
| PM_2.5_ (5 μg/m^3^) | 1.47 (0.89, 2.43) | 1.01 (0.72, 1.40) | 0.028 (-0.042, 0.135) |
| NO_2_ (5 ppb) | 0.83 (0.38, 1.80) | 0.92 (0.63, 1.35) | -0.017 (-0.116, 0.056) |
| O_3_ (5 ppb) | 0.97 (0.63, 1.51) | 0.83 (0.66, 1.05) | -0.002 (-0.049, 0.030) |
| SO_2_ (0.5 ppb) | 1.08 (0.71, 1.64) | 0.91 (0.70, 1.17) | 0.004 (-0.018, 0.027) |
| Pre-implantation (1-5 days after sample) | |  |  |
| PM_2.5_ (5 μg/m^3^) | 1.64 (0.94, 2.85) | 1.07 (0.83, 1.39) | 0.031 (-0.014, 0.094) |
| NO_2_ (5 ppb) | 0.60 (0.24, 1.52) | 1.01 (0.70, 1.46) | -0.038 (-0.117, 0.018) |
| O_3_ (5 ppb) | 0.91 (0.57, 1.47) | 0.95 (0.76, 1.19) | -0.005 (-0.038, 0.020) |
| SO_2_ (0.5 ppb) | 1.10 (0.78, 1.56) | 0.90 (0.70, 1.16) | 0.039 (-0.001, 0.090) |
| Implantation (6-10 days after sample) | |  |  |
| PM_2.5_ (5 μg/m^3^) | 0.92 (0.36, 2.38) | 1.00 (0.74, 1.36) | -0.003 (-0.057, 0.046) |
| NO_2_ (5 ppb) | 1.09 (0.44, 2.70) | 0.79 (0.53, 1.16) | 0.021 (-0.020, 0.057) |
| O_3_ (5 ppb) | 1.12 (0.70, 1.79) | 0.77 (0.62, 0.96) | 0.025 (0.003, 0.043) |
| SO_2_ (0.5 ppb) | 0.99 (0.64, 1.54) | 0.88 (0.67, 1.16) | 0.014 (-0.007, 0.036) |
| Post-implantation (11-14 days after sample) | |  |  |
| PM_2.5_ (5 μg/m^3^) | 0.94 (0.42, 2.09) | 0.75 (0.53, 1.07) | 0.014 (-0.040, 0.057) |
| NO_2_ (5 ppb) | 0.97 (0.44, 2.17) | 1.43 (0.98, 2.09) | -0.025 (-0.086, 0.027) |
| O_3_ (5 ppb) | 1.10 (0.70, 1.72) | 1.09 (0.88, 1.35) | 0.003 (-0.021, 0.025) |
| SO_2_ (0.5 ppb) | 1.17 (0.88, 1.56) | 0.88 (0.68, 1.13) | 0.032 (0.000, 0.083) |

PM_2.5_: Particulate matter <2.5 microns; NO_2_: Nitrogen dioxide; O_3_: Ozone; SO_2_: Sulfur dioxide. All models adjust for male age, season, and income. Multipollutant models additionally adjust for co-pollutants PM_2.5_, NO_2_, O_3_, and SO_2_.

Table S15. Association of air pollution and pregnancy in intrauterine insemination (IUI) cycles by baseline semen quality parameters <WHO 5^th^ percentile thresholds (n=1,158).

|  | Pregnancy by baseline semen quality parameters <WHO 5% thresholds | | |
| --- | --- | --- | --- |
|  | Multipollutant | | |
|  | <WHO 5^th^ %ile | ≥WHO 5^th^ %ile | RERI |
|  | RR (95% CI) | RR (95% CI) | Estimate (95% CI) |
| Spermatogenesis (0-74 days before sample) |  |  |  |
| PM_2.5_ (5 μg/m^3^) | 1.29 (0.76, 2.21) | 1.27 (0.80, 2.01) | -0.080 (-0.184, 0.018) |
| NO_2_ (5 ppb) | 0.60 (0.35, 1.01) | 0.89 (0.54, 1.45) | 0.012 (-0.126, 0.111) |
| O_3_ (5 ppb) | 0.67 (0.48, 0.93) | 0.76 (0.55, 1.05) | -0.005 (-0.089, 0.055) |
| SO_2_ (0.5 ppb) | 1.00 (0.78, 1.28) | 0.91 (0.70, 1.19) | 0.009 (-0.029, 0.051) |
| Mitosis (59-74 days before sample) |  |  |  |
| PM_2.5_ (5 μg/m^3^) | 1.45 (1.03, 2.06) | 1.24 (0.88, 1.75) | 0.004 (-0.068, 0.088) |
| NO_2_ (5 ppb) | 0.62 (0.39, 0.98) | 0.78 (0.51, 1.19) | -0.019 (-0.134, 0.073) |
| O_3_ (5 ppb) | 0.75 (0.58, 0.97) | 0.79 (0.62, 1.00) | -0.014 (-0.078, 0.034) |
| SO_2_ (0.5 ppb) | 1.06 (0.86, 1.30) | 0.93 (0.73, 1.19) | 0.021 (-0.014, 0.060) |
| Meiosis I+II (33-58 days before sample) |  |  |  |
| PM_2.5_ (5 μg/m^3^) | 1.18 (0.80, 1.75) | 1.19 (0.81, 1.74) | -0.043 (-0.118, 0.030) |
| NO_2_ (5 ppb) | 0.70 (0.45, 1.10) | 0.94 (0.60, 1.48) | 0.026 (-0.079, 0.111) |
| O_3_ (5 ppb) | 0.74 (0.56, 0.99) | 0.80 (0.60, 1.06) | 0.011 (-0.048, 0.058) |
| SO_2_ (0.5 ppb) | 0.98 (0.75, 1.26) | 0.88 (0.67, 1.15) | 0.016 (-0.019, 0.051) |
| Spermiogenesis (11-32 days before sample) |  |  |  |
| PM_2.5_ (5 μg/m^3^) | 1.07 (0.69, 1.66) | 0.97 (0.65, 1.44) | -0.016 (-0.091, 0.058) |
| NO_2_ (5 ppb) | 0.74 (0.46, 1.19) | 1.17 (0.77, 1.79) | -0.050 (-0.158, 0.034) |
| O_3_ (5 ppb) | 0.78 (0.59, 1.05) | 0.92 (0.69, 1.22) | -0.028 (-0.096, 0.023) |
| SO_2_ (0.5 ppb) | 0.95 (0.74, 1.22) | 0.94 (0.75, 1.19) | 0.000 (-0.046, 0.046) |
| Spermiation (0-10 days before sample) |  |  |  |
| PM_2.5_ (5 μg/m^3^) | 0.97 (0.66, 1.43) | 1.10 (0.82, 1.48) | -0.069 (-0.130, -0.005) |
| NO_2_ (5 ppb) | 0.75 (0.47, 1.18) | 1.03 (0.71, 1.49) | 0.038 (-0.024, 0.095) |
| O_3_ (5 ppb) | 0.79 (0.61, 1.02) | 0.87 (0.69, 1.10) | 0.016 (-0.019, 0.044) |
| SO_2_ (0.5 ppb) | 1.02 (0.81, 1.28) | 0.88 (0.67, 1.16) | 0.008 (-0.035, 0.051) |
| Ovulation (0-5 days before sample) |  |  |  |
| PM_2.5_ (5 μg/m^3^) | 1.12 (0.80, 1.58) | 1.07 (0.80, 1.43) | -0.047 (-0.101, 0.005) |
| NO_2_ (5 ppb) | 0.73 (0.47, 1.12) | 1.08 (0.74, 1.56) | 0.026 (-0.037, 0.082) |
| O_3_ (5 ppb) | 0.79 (0.63, 1.01) | 0.89 (0.71, 1.12) | 0.014 (-0.020, 0.041) |
| SO_2_ (0.5 ppb) | 1.02 (0.82, 1.27) | 0.81 (0.60, 1.10) | 0.028 (-0.010, 0.069) |
| Pre-implantation (1-5 days after sample) |  |  |  |
| PM_2.5_ (5 μg/m^3^) | 1.09 (0.78, 1.51) | 1.01 (0.76, 1.33) | -0.029 (-0.075, 0.021) |
| NO_2_ (5 ppb) | 0.80 (0.51, 1.27) | 1.15 (0.80, 1.64) | 0.028 (-0.056, 0.098) |
| O_3_ (5 ppb) | 0.87 (0.69, 1.11) | 1.00 (0.81, 1.24) | 0.010 (-0.026, 0.040) |
| SO_2_ (0.5 ppb) | 0.98 (0.79, 1.22) | 0.93 (0.72, 1.20) | -0.004 (-0.042, 0.035) |
| Implantation (6-10 days after sample) |  |  |  |
| PM_2.5_ (5 μg/m^3^) | 1.04 (0.69, 1.57) | 1.08 (0.83, 1.39) | -0.028 (-0.084, 0.032) |
| NO_2_ (5 ppb) | 0.66 (0.41, 1.06) | 1.08 (0.76, 1.53) | -0.021 (-0.098, 0.049) |
| O_3_ (5 ppb) | 0.82 (0.64, 1.04) | 0.92 (0.74, 1.13) | 0.005 (-0.034, 0.040) |
| SO_2_ (0.5 ppb) | 0.99 (0.78, 1.25) | 0.86 (0.65, 1.13) | 0.021 (-0.012, 0.060) |
| Post-implantation (11-14 days after sample) |  |  |  |
| PM_2.5_ (5 μg/m^3^) | 0.99 (0.68, 1.44) | 1.00 (0.79, 1.26) | -0.015 (-0.072, 0.040) |
| NO_2_ (5 ppb) | 0.90 (0.59, 1.37) | 1.39 (0.99, 1.96) | -0.042 (-0.116, 0.024) |
| O_3_ (5 ppb) | 0.96 (0.76, 1.20) | 1.14 (0.93, 1.40) | -0.014 (-0.047, 0.015) |
| SO_2_ (0.5 ppb) | 1.04 (0.85, 1.28) | 0.91 (0.70, 1.17) | 0.017 (-0.026, 0.064) |

PM_2.5_: Particulate matter <2.5 microns; NO_2_: Nitrogen dioxide; O_3_: Ozone; SO_2_: Sulfur dioxide. All models adjust for male age, season, and income. Multipollutant models additionally adjust for co-pollutants PM_2.5_, NO_2_, O_3_, and SO_2_.

Table S16. Association of air pollution and live birth in intrauterine insemination (IUI) cycles by baseline semen quality parameters <WHO 5^th^ percentile thresholds (n=1,158).

|  | Live birth by baseline semen quality parameters <WHO 5% thresholds | | |
| --- | --- | --- | --- |
|  | Multipollutant | | |
|  | <WHO 5^th^ %ile | <WHO 5^th^ %ile | <WHO 5^th^ %ile |
|  | RR (95% CI) | RR (95% CI) | RR (95% CI) |
| Spermatogenesis (0-74 days before sample) |  |  |  |
| PM_2.5_ (5 μg/m^3^) | 1.38 (0.75, 2.55) | 1.01 (0.53, 1.93) | -0.008 (-0.068, 0.057) |
| NO_2_ (5 ppb) | 0.80 (0.43, 1.48) | 0.92 (0.47, 1.78) | 0.005 (-0.090, 0.085) |
| O_3_ (5 ppb) | 0.78 (0.52, 1.15) | 0.77 (0.51, 1.19) | -0.008 (-0.062, 0.034) |
| SO_2_ (0.5 ppb) | 0.98 (0.74, 1.30) | 0.92 (0.64, 1.31) | 0.008 (-0.025, 0.044) |
| Mitosis (59-74 days before sample) |  |  |  |
| PM_2.5_ (5 μg/m^3^) | 1.43 (0.95, 2.14) | 1.22 (0.78, 1.92) | 0.010 (-0.035, 0.060) |
| NO_2_ (5 ppb) | 0.82 (0.48, 1.40) | 0.78 (0.45, 1.35) | 0.004 (-0.057, 0.064) |
| O_3_ (5 ppb) | 0.87 (0.65, 1.18) | 0.79 (0.58, 1.07) | -0.002 (-0.034, 0.030) |
| SO_2_ (0.5 ppb) | 1.03 (0.82, 1.30) | 0.94 (0.69, 1.29) | 0.010 (-0.023, 0.039) |
| Meiosis I+II (33-58 days before sample) |  |  |  |
| PM_2.5_ (5 μg/m^3^) | 1.15 (0.72, 1.85) | 0.93 (0.54, 1.60) | 0.001 (-0.064, 0.057) |
| NO_2_ (5 ppb) | 0.91 (0.54, 1.53) | 1.12 (0.60, 2.09) | 0.006 (-0.067, 0.073) |
| O_3_ (5 ppb) | 0.82 (0.59, 1.15) | 0.86 (0.59, 1.25) | -0.004 (-0.043, 0.026) |
| SO_2_ (0.5 ppb) | 0.95 (0.71, 1.29) | 0.87 (0.60, 1.26) | 0.008 (-0.024, 0.039) |
| Spermiogenesis (11-32 days before sample) |  |  |  |
| PM_2.5_ (5 μg/m^3^) | 1.11 (0.67, 1.84) | 0.88 (0.51, 1.52) | 0.012 (-0.043, 0.069) |
| NO_2_ (5 ppb) | 0.91 (0.53, 1.57) | 1.09 (0.62, 1.94) | -0.042 (-0.141, 0.030) |
| O_3_ (5 ppb) | 0.86 (0.61, 1.21) | 0.90 (0.62, 1.31) | -0.027 (-0.080, 0.013) |
| SO_2_ (0.5 ppb) | 0.94 (0.71, 1.25) | 0.96 (0.70, 1.33) | 0.009 (-0.029, 0.046) |
| Spermiation (0-10 days before sample) |  |  |  |
| PM_2.5_ (5 μg/m^3^) | 0.92 (0.58, 1.46) | 1.03 (0.68, 1.56) | -0.039 (-0.086, 0.009) |
| NO_2_ (5 ppb) | 0.89 (0.52, 1.50) | 0.98 (0.59, 1.64) | 0.034 (-0.022, 0.087) |
| O_3_ (5 ppb) | 0.83 (0.61, 1.13) | 0.91 (0.67, 1.23) | 0.002 (-0.027, 0.026) |
| SO_2_ (0.5 ppb) | 1.00 (0.77, 1.30) | 0.86 (0.59, 1.27) | 0.001 (-0.036, 0.037) |
| Ovulation (0-5 days before sample) |  |  |  |
| PM_2.5_ (5 μg/m^3^) | 1.03 (0.70, 1.53) | 1.15 (0.79, 1.67) | -0.036 (-0.078, 0.011) |
| NO_2_ (5 ppb) | 0.91 (0.55, 1.49) | 0.94 (0.58, 1.52) | 0.034 (-0.017, 0.082) |
| O_3_ (5 ppb) | 0.83 (0.63, 1.11) | 0.90 (0.67, 1.19) | 0.004 (-0.024, 0.027) |
| SO_2_ (0.5 ppb) | 0.99 (0.77, 1.27) | 0.83 (0.56, 1.23) | 0.012 (-0.012, 0.037) |
| Pre-implantation (1-5 days after sample) |  |  |  |
| PM_2.5_ (5 μg/m^3^) | 1.07 (0.73, 1.57) | 1.14 (0.82, 1.58) | -0.040 (-0.084, 0.001) |
| NO_2_ (5 ppb) | 0.84 (0.49, 1.41) | 1.13 (0.71, 1.79) | 0.033 (-0.034, 0.089) |
| O_3_ (5 ppb) | 0.86 (0.65, 1.14) | 1.10 (0.84, 1.45) | -0.004 (-0.035, 0.023) |
| SO_2_ (0.5 ppb) | 0.93 (0.71, 1.22) | 0.96 (0.69, 1.34) | -0.015 (-0.052, 0.021) |
| Implantation (6-10 days after sample) |  |  |  |
| PM_2.5_ (5 μg/m^3^) | 0.83 (0.48, 1.43) | 1.07 (0.74, 1.54) | -0.020 (-0.071, 0.025) |
| NO_2_ (5 ppb) | 0.78 (0.45, 1.35) | 0.89 (0.56, 1.44) | -0.010 (-0.070, 0.042) |
| O_3_ (5 ppb) | 0.80 (0.60, 1.06) | 0.84 (0.64, 1.10) | -0.008 (-0.044, 0.023) |
| SO_2_ (0.5 ppb) | 0.93 (0.70, 1.24) | 0.83 (0.56, 1.23) | 0.010 (-0.018, 0.038) |
| Post-implantation (11-14 days after sample) |  |  |  |
| PM_2.5_ (5 μg/m^3^) | 0.60 (0.33, 1.09) | 0.86 (0.60, 1.24) | -0.036 (-0.085, 0.007) |
| NO_2_ (5 ppb) | 1.10 (0.66, 1.82) | 1.56 (0.99, 2.46) | -0.014 (-0.078, 0.042) |
| O_3_ (5 ppb) | 0.97 (0.74, 1.28) | 1.25 (0.96, 1.63) | -0.018 (-0.047, 0.004) |
| SO_2_ (0.5 ppb) | 1.02 (0.80, 1.31) | 0.92 (0.66, 1.28) | 0.001 (-0.044, 0.039) |

PM_2.5_: Particulate matter <2.5 microns; NO_2_: Nitrogen dioxide; O_3_: Ozone; SO_2_: Sulfur dioxide. All models adjust for male age, season, and income. Multipollutant models additionally adjust for co-pollutants PM_2.5_, NO_2_, O_3_, and SO_2_.

Figure S1. Flowchart for inclusion/exclusion for participants and IUI cycles from original FAZST cohort.

Figure S2. Windows of susceptibility and developmental stages during spermatogenesis

Figure S3. Pearson Correlation Plot: Criteria Air Pollutants (Overall, Jan-Dec) 74-Day Spermatogenesis Window *(n*=588 couples). PM_2.5_: particulate matter <2.5 microns; NO_2_: nitrogen dioxide; O_3_: ozone: SO_2_: sulfur dioxide

**Correlation plot legend**

Strong positive: 0.6 – 0.8

Moderate positive: 0.4 – 0.6

Low positive: 0.1 – 0.4

Low negative: -0.1 – -0.4

Moderate negative: -0.4 – 0.6

Strong negative: -0.6 – -.0.8

Figure S4. Pearson Correlation Plot: Criteria Air Pollutants 74-Day Spermatogenesis Window (Warm Season, Apr-Sept) *(n*=305 couples). PM_2.5_: particulate matter <2.5 microns; NO_2_: nitrogen dioxide; O_3_: ozone: SO_2_: sulfur dioxide.

**Correlation plot legend**

Strong positive: 0.6 – 0.8

Moderate positive: 0.4 – 0.6

Low positive: 0.1 – 0.4

Low negative: -0.1 – -0.4

Moderate negative: -0.4 – 0.6

Strong negative: -0.6 – -.0.8

Figure S5. Pearson Correlation Plot: Criteria Air Pollutants, 74-Day Spermatogenesis Window (Cold Season, Oct-March) *(n*=283 couples). PM_2.5_: particulate matter <2.5 microns; NO_2_: nitrogen dioxide; O_3_: ozone: SO_2_: sulfur dioxide

**Correlation plot legend**

Strong positive: 0.6 – 0.8

Moderate positive: 0.4 – 0.6

Low positive: 0.1 – 0.4

Low negative: -0.1 – -0.4

Moderate negative: -0.4 – 0.6

Strong negative: -0.6 – -.0.8

Figure S6. Relative weights for each air pollutant in quantile g-computation models by window of exposure and outcome. PM_2.5_: Particulate matter <2.5 microns; NO_2_: Nitrogen dioxide; O_3_: Ozone; SO_2_: Sulfur dioxide (n=1,176 for pregnancy and live birth and n=127 for pregnancy loss).

Figure S7. Relationship between exposure to ambient air pollutants across windows of susceptibility during ovulation and implantation and outcomes of intrauterine insemination cycles for single-pollutant, multipollutant, and mixture models (n=1,176 for pregnancy and live birth and n=127 for pregnancy loss). Particulate matter <2.5 microns (dark blue, PM_2.5_) modeled per 5 μg/m^3^, nitrogen dioxide (red, NO_2_) and ozone (light blue, O_3_) modeled per 5 ppb, sulfur dioxide (pink, SO_2_) modeled per 0.5 ppb, and mixture (green) modeled per decile increase. All models adjust for men’s age, season, and household income, and multipollutant models jointly adjust for PM_2.5_, NO_2_, O_3_, and SO_2_.

Figure S8. Relationship between exposure to ambient air pollutants across windows of susceptibility during ovulation and implantation and outcomes of intrauterine insemination cycles for multipollutant, and mixture models by warm (Apr. 1 – Sept. 30) versus cold (Oct.1 – Mar. 31) season (n=1,176). Particulate matter <2.5 microns (dark blue, PM_2.5_) modeled per 5 μg/m^3^, nitrogen dioxide (red, NO_2_) and ozone (light blue, O_3_) modeled per 5 ppb, sulfur dioxide (pink, SO_2_) modeled per 0.5 ppb, and mixture (black) modeled per decile increase. All models adjusted for men’s age, season, and household income, and multipollutant models jointly adjust for PM_2.5_, NO_2_, O_3_, and SO_2_.
